# Halogen Bonds or Not? Reassessing Noncovalent Interactions in Crystals of Periodate Anion from the Cambridge Structural Database

**DOI:** 10.3390/molecules31122153

**Published:** 2026-06-18

**Authors:** Arpita Varadwaj, Pradeep R. Varadwaj, Helder M. Marques, Ireneusz Grabowski, Koichi Yamashita, Mohd. Mudassir Husain

**Affiliations:** 1 Department of Chemical System Engineering, School of Engineering, University of Tokyo, 7-3-1, Tokyo 113-8656, Japan; 2 Institute of Physics, Faculty of Physics, Astronomy, and Informatics, Nicolaus Copernicus University in Toruń, 87-100 Toruń, Poland; ig@fizyka.umk.pl; 3 Molecular Sciences Institute, School of Chemistry, University of the Witwatersrand, Johannesburg 2050, South Africa; 4 Institute of Advanced Studies, Nicolaus Copernicus University in Toruń, Ul. Wileńska 4, 87-100 Toruń, Poland; 5 Department of Applied Sciences and Humanities, Faculty of Engineering and Technology, Jamia Millia Islamia, New Delhi 110025, India

**Keywords:** anion–anion non-covalent interactions, halogen-centered directional interactions, σ-hole centered interactions, physical chemistry and chemical physics, MESP, QTAIM, IGMH and SAPT analyses, interaction topology, crystallography, environmental effects

## Abstract

This study examines a series of organic–inorganic crystal structures containing the periodate anion (IO_4_^−^) to clarify the nature of the anion–anion interactions that are frequently referred to as halogen bonds. Our analysis demonstrates that, in many cases, IO_4_^−^ does not develop an electrophilic σ-hole on the iodine center, even in the presence of organic cations, and therefore cannot reliably function as a halogen-bond donor. In its discrete (0D) form, the anion retains its character as a Lewis base. In crystal structures where extended architectures are observed—such as one-dimensional chains, two-dimensional layers, or three-dimensional cage-like assemblies—these structures arise predominantly from strong coulombic interactions with surrounding cations, as the interaction between the anions is intrinsically repulsive in the gas phase. Hydrogen bonding, together with other noncovalent interactions including chalcogen, tetrel, and/or pnictogen bonding, plays a dominant role in stabilizing the anionic arrangements and governing their structural organization.

## 1. Introduction

Noncovalent interactions are widely recognized as the chemical glue that governs molecular recognition, self-assembly, and the formation of functional crystalline materials [1,2,3]. Beyond classical hydrogen bonding [4] and the increasingly studied σ-hole driven interactions such as halogen [5,6], chalcogen [7], pnictogen [8,9], and tetrel bonding [10], attention has recently turned to a more unusual class of contact: anti-electrostatic interactions between like-charged species [11,12,13,14]. These interactions are counterintuitive because coulombic considerations predict strong repulsion between similarly charged ions, yet many experimental and computational studies have demonstrated that such species can form well-defined dimers, oligomers, and extended architectures, driven by halogen bonding [11,13].

Close contacts between molecular entities (e.g., anions) have been interpreted as arising from polarization, charge-transfer, dispersion, and environment-mediated stabilization [14,15,16], leading to the emergence of ordered structures in both molecular clusters and crystalline solids [17,18,19,20]. These findings challenge the conventional electrostatic picture of intermolecular interactions and highlight the need for a rigorous analysis of the physical origins of the apparent attractive contacts between like-charged ions.

Halogen oxyanions, especially XO_4_^−^ (X = Cl, Br, I) [13,21,22,23,24,25], provide a compelling platform for examining anti-electrostatic interactions [13,26,27]. Many crystal structures in the Cambridge Structural Database (CSD) [28,29] reveal the occurrence of X···O contacts between perchlorate, perbromate, and periodate anions, often forming dimers, chains, or higher-order oligomers in the solid state. These contacts frequently appear directional, sometimes aligning along extensions of X–O bonds (e.g., *r*(I···O) = 4.129 and (∠O–I···O = 165.2° in [C_6_H_14_NO_2_^+^, IO_4_^−^] (CSD ref: IHEGUZ)) [21], and have therefore been interpreted as σ-hole driven halogen bonds [13,26]. Several computational investigations [13,26] have further suggested that such (XO_4_^−^)_n_, or (XO_3_^−^)_n_, assemblies in crystals may be stabilized by lone-pair (O) → σ* (X–O) charge transfer or polarization-assisted attraction [13,30], despite the formally repulsive electrostatic interaction between anions. In extended crystals, these motifs propagate into one-dimensional chains, two-dimensional sheets, or pseudo-three-dimensional frameworks, mediated by counterions that organize the anionic sublattice. Consequently, the existence of XO_4_^−^ or XO_3_^−^ dimers and their oligomers has been invoked as evidence for halogen bonding between anions [13,30], although the intrinsic nature of intermolecular stability leading to these assemblies and their physical origin yet to be fully understood.

What are halogen bonds?

A halogen bond (HaB) develops in a chemical system when a net attractive engagement occurs between an electron-density-deficient (electrophilic) region on the electrostatic surface of a halogen atom in a molecular entity and a close-lying electron-density-rich (nucleophilic) region on the electrostatic surface of the same or another identical or different molecular entity [5].

A majority of dimer models, as discussed in many research articles [3,31,32,33,34], have focused on the implications of σ-holes in covalently bonded halogen derivatives. These σ-holes enable halogen atoms to engage directionally with electron-rich sites, sustaining halogen bonding, which is often associated with the positive character of the σ-hole acting as a halogen-bond donor.

A σ-hole is an electron density-deficient region on the surface of an atom A in R–A, located along the extension of the covalent bond opposite to R, where R represents the remainder of the molecular entity [3]. It can be either positive or negative [1,35]. While both characteristics are determined by the maximum value of the electrostatic potential, $V_{S,max}$, the sign of $V_{S,max}$ defines its nature. For example, when $V_{S,max}>0$, the region corresponds to what is commonly referred to as a σ-hole of electrophilic character. In contrast, when $V_{S,max}<0$, the region should be described as a negative σ-hole, provided that it appears along the extension of the R–A bond axis. Examples of positive σ-holes are readily visualized on the electrostatic potential surfaces of diatomic halogen molecules, X_2_ (X = F, Cl, Br, I). A negative σ-hole on fluorine is observed along the extension of the C–F bond in H_3_C–F and F_5_C_6_–F, respectively.

However, alternative interpretations continue to exist regarding the relationship between electrostatic potential and the recognition of halogen-centered interactions. Some viewpoints restrict σ-hole-driven interactions exclusively to cases where the interacting halogen exhibits a strictly positive electrostatic potential along the extension of the covalent bond, while others consider the term σ-hole itself to refer only to positive regions of electrostatic potential. Within such an interpretation, interactions involving fluorine atoms associated with negative maximum of potential [35], or contacts formed through lateral belt regions [5], were argued to be excluded from the halogen-bonding framework. Nevertheless, several recent studies have shown that directional halogen-centered interactions may persist even in systems where the electrostatic potential remains negative, particularly in highly polarized or charged environments where polarization, dispersion, and many-body effects contribute significantly to stabilization [26,27]. Related examples have also been discussed for fluorinated systems, including cases in which fluorine may participate through anisotropic belt regions rather than conventional positive σ-hole regions that are halogen bonds (vide infra) [5]. These observations suggest that the sign of electrostatic potential is a valuable tool to fully capture the diversity and physical complexity of halogen-centered intermolecular interactions.

As indicated above, halogen bonds are not always σ-hole-centered interactions. When the interaction occurs along the extension of a covalently bonded halogen atom and the halogen donates an electrophilic σ-hole, the interaction is a σ-hole-centered halogen bond. However, there are instances in which the p- or π-electron density on the halogen surface participates in bonding with a negative site; these are also halogen bonds but correspond to orthogonal halogen bonding [36,37]. Instances are also seen where the σ-hole is not involved, and the halogen interacts laterally through regions where lone-pair electron density typically resides, as observed for some halogenated cations. These interactions therefore represent non-σ-hole-centered halogen bonding [5,6].

4-Acetoamido-3-(1-acetyl-2-(2,6-dichlorobenzylidene)hydrazine)-1,2,4-triazole (C_13_H_12_Cl_2_N_6_O_2_) (CSD ref. AABHTZ) [38,39] is a notable example of a system in which the lateral side of a covalently bonded chlorine atom interacts with the nucleophilic oxygen of the O=C fragment, forming a Cl···O close contact (Cl···O = 3.290 Å). This is an intramolecular interaction. Considering directionality, the C=O···Cl angle (∠C=O···Cl = 177.3°) is more linear than the C–Cl···O angle (∠C–Cl···O = 109.1°). Despite this directionality, the interaction should be recognized as a halogen bond rather than a chalcogen bond [7], because the electrophilic chlorine atom (not that on the σ-hole) engages with a nucleophilic site.

A further question arises when negative σ-holes on halogen atoms in molecular entities participate in directional halogen-centered close contacts [35], as observed between anions in crystals [13]. These interactions do not strictly conform to the conventional definition of halogen bonding, which assumes an electrophilic region on the halogen interacting with a nucleophilic site [5,6]. Instead, both interacting sites may be electron-rich, and the electrostatic component is often repulsive when modeled in the gas phase where no external constraint is operative on the interacting monomers. Nevertheless, the interactions remain directional and halogen-centered, reflecting the anisotropy of the electron density around the covalently bonded halogen derivative.

The same issue arises when such systems are modeled in solution without counterions [13,26,40]. Even in the absence of cations, directional halogen-centered contacts between anions may persist, stabilized by solvent-mediated induction and dispersion contributions despite anti-electrostatic components. These interactions therefore resemble halogen-centered non-covalent interactions geometrically but differ from conventional (electrophilic) σ-hole-driven halogen bonds in their physical origin.

What terminology is appropriate for intermolecular interactions in which negative σ-holes on covalently bonded halogen atoms give rise to directional, halogen-centered close contacts between anions in crystal structures? Furthermore, how should these interactions be described in the presence of cations or under solution-phase conditions? Do these contacts truly represent halogen bonding, or are they more appropriately referred to as alternative noncovalent interactions, such as “anti-electrostatic” halogen bonds?

Herein, we revisit selected crystal structures to address these questions and to assess whether the presence of a cation indeed polarizes the anion to induce an electrophilic σ-hole on the halogen, thereby promoting anion–anion contacts in IO_4_^−^ assemblies via σ-holes on the covalently bonded iodine atom. We examine several ion pairs in solution using density functional theory, employing the Solvation Model based on Density (SMD) [41] with water as the solvent. The Quantum Theory of Atoms in Molecules (QTAIM) [42], Molecular Electrostatic Surface Potential (MESP) [43,44,45,46], and the Independent Gradient Model based on Hirshfeld partitioning (IGMH) [47] are used to characterize the chemical interactions. In selected cases, Symmetry-Adapted Perturbation Theory (SAPT) [48,49] is also applied to elucidate the thermodynamic nature of the intermolecular interactions between the species.

What follows summarizes the bonding pattern of a negative σ-hole in a simple system, such as H_3_C–F in the gas phase, and then compares it with analogous interactions in solution and crystalline phases, where negative σ-holes contribute to the cation-mediated self-assembly of anions.

## 2. Beyond Classical σ-Hole Bonding: Negative σ-Holes and Weak Anion–Anion Interactions

**Figure 1:**
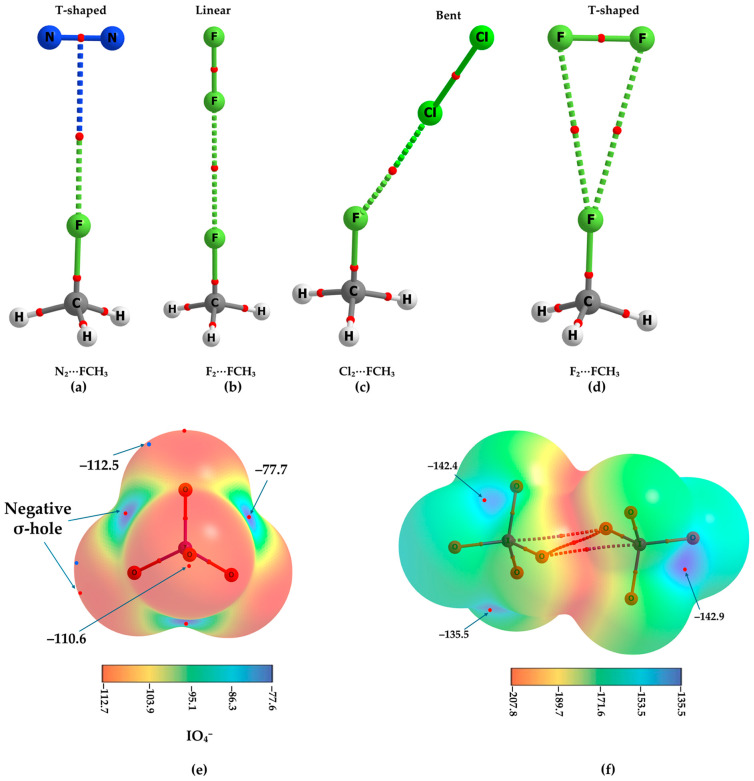
QTAIM molecular graphs of the complexes of CH_3_F with (**a**) N_2_, (**b**) F_2_, (**c**) Cl_2_, and (**d**) F_2_. Bond paths are shown as solid and dashed lines in atom colors, and bond critical points are represented as small red spheres along the bond paths. (**e**) and (**f**): QTAIM molecular graph superimposed on the MESP plots of IO_4_^−^ and (IO_4_^−^)_2_, respectively, where selected maxima (small red circles) and minima (small blue circles) of the electrostatic potential (kcal mol^−1^) are indicated by arrows.

Fluorine in CH_3_F exhibits a negative σ-hole (*V_S,max_* = −22.2 kcal mol^−1^) along the C–F bond extension yet retains clear directional bonding capability toward electrophilic regions. In complexes with N_2_ and F_2_, interactions align along this negative σ-hole, although their nature depends on the interacting partner. For example, the T-shaped N_2_⋯FCH_3_ complex is best described as a π-centered pnictogen bond (Figure 1a), whereas the linear F_2_⋯FCH_3_ structure represents a σ-hole-driven halogen bond (Figure 1b). Similarly, the bent Cl_2_⋯FCH_3_ geometry (Figure 1c) arises from interaction between the fluorine negative lateral side and the electrophilic σ-hole on Cl of Cl_2_, forming a linear σ-hole-centered halogen bond. The T-shaped F_2_⋯FCH_3_ geometry in Figure 1d arises from interaction between the fluorine negative σ-hole and the electrophilic belt of F_2_, forming a *p*-belt-driven halogen bond. SAPT analysis shows that dispersion dominates stabilization in all cases.

For XO_4_^−^ (X = Cl, Br, I) anions, negative σ-holes occur on both the halogen and oxygen atoms, indicating their ability to participate in directional noncovalent interactions. The nature of the molecular electrostatic surface potential (MESP) of IO_4_^−^ is illustrated in Figure 1e, showing that oxygen is more nucleophilic than iodine when their σ-hole potentials are compared. The strength of interaction between anions, whether identical or different, depends on the polarizability of the halogen center; thus, iodine-containing species are expected to exhibit stronger interactions than their lighter halogen counterparts when the interaction energies of (XO_4_^−^)_2_ are compared.

The (IO_4_^−^)_2_ dimer extracted from the crystal structure was energy-minimized using the SMD model, and its QTAIM molecular graph, superimposed on the MESP plot, is shown in Figure 1f. The molecular graph reveals three bond paths, indicating the presence of two O···I and one O···O close contacts. The former has a Type-II topology, whereas the latter corresponds to a Type-I interaction [1]. Although the O···I contacts are quasi-linear and centered on negative σ-holes, they cannot be classified as halogen bonds, as they do not satisfy the fundamental requirement of an electrophilic (positive) σ-hole on the covalently bonded iodine. The binding energy of the dimer is −1.65 kcal mol^−1^.

The apparent stabilization of these assemblies originates from solvent screening, polarization induced by neighboring ions, and crystal packing effects that mitigate coulombic repulsion and allow weaker induction and dispersion contributions to dictate the geometry. These findings have important implications for interpreting directional contacts in crystalline materials, emphasizing that the presence of short, quasi-linear X···O interactions in XO_4_^−^ dimers and oligomers does not necessarily imply intrinsic halogen bonding, but may instead reflect environment-stabilized anti-electrostatic association. Further details will be reported elsewhere.

## 3. Geometric Features of Some Organic–Inorganic Crystal Systems

Our survey of the CSD (version 6.01) identified 58 crystal structures, of which 26 contain the molecular form of the periodate anion acting as a counterion. It is difficult to determine whether it functions as a halogen-bond donor in each case without detailed computational analysis; however, it commonly operates as a hydrogen-bond acceptor, or more generally as a Lewis base, toward a variety of organic cations, including bis(tetramethyltetrathiafulvalenium) (CSD ref: DOFNAN01) [50], as well as metal cations such as alkali or alkaline-earth ions (e.g., Rb^+^ and K^+^) [27].

The search for IO_4_···O–X contacts was carried out using an I···O distance criterion of 2.0–4.0 Å and an O–I···O angle range of 140–180°, resulting in 49 individual close contacts extracted from 16 unique crystal structures containing the anion (CSD refcodes: BEKNIS, BEKNOY, BEKNUE, BEKPAM, HOHMOG05, IHEHAG, JOJYOY, JOJZAL, LANNEU, TEJBET, TEJBET01, TEJBIX, TEJBIX01, WEMSUD, YIVYUB, and YOFBAA). Only crystal structures with R-factor ≤ 0.05, free of reported errors and based on single-crystal determinations, were included. Each refcode contributes between one and five independent geometrical entries, indicating that the observed interaction motif is reproducible across multiple crystalline environments rather than being confined to a single structure type.

The I···O distances span a range from 3.105 Å to 3.966 Å, with most contacts clustering in the 3.3–3.8 Å region, consistent with long-range, weakly attractive or packing-induced interactions rather than covalent or strongly bound motifs. The angular distribution is relatively narrow, with O–I···O angles ranging from 140° to 179°, and a pronounced concentration near linear geometries (160–180°), indicating a strong directional bias along the O–I bond extension. Tables S1 and S2 in the Supplementary Materials provide the CSD refcodes and the corresponding intermolecular geometries (*r*(I···O) and ∠O–I···O) for the crystal structures analyzed, together with statistical summaries of the dataset, including mean values.

The intermolecular interactions between the anions in some crystals lead to the formation of architectures spanning 1D, 2D, and 3D networks. In the latter case, the network serves to host organic or inorganic cations, whereas in 2D systems, the cations act as spacers separating the layers. Representative examples for each dimensionality are discussed below.

**Figure 2:**
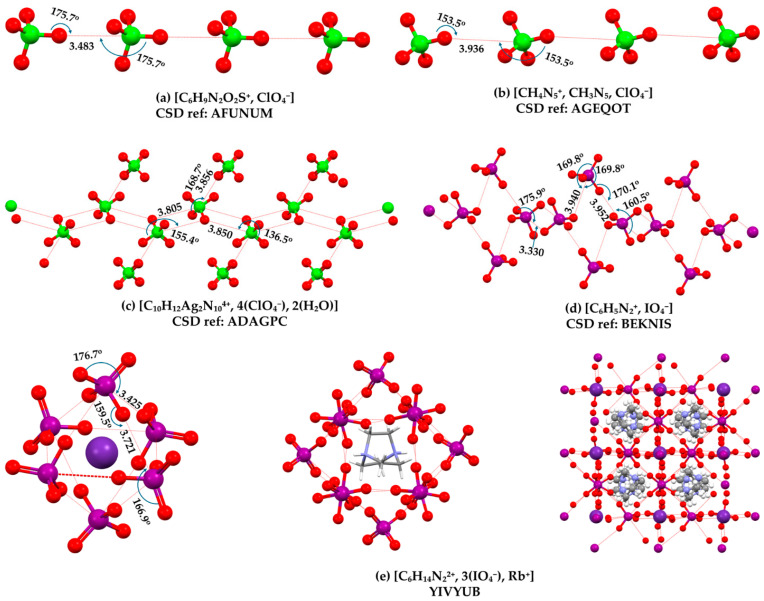
Illustration of selected crystal structures in which the XO_4_^−^ (X = Cl, I) anion assembles into (**a**,**b**) one-dimensional, (**c**,**d**) two-dimensional, and (**e**) three-dimensional architectures in the presence of organic and/or inorganic cations. Selected intermolecular distances and bond angles are given in Å and °, respectively. The organic cations are omitted in (**a**–**d**) for clarity. Dashed lines represent close contacts. Atom labels: Cl—green balls; O—red balls; I—purple balls.

[C_6_H_9_N_2_O_2_S^+^, ClO_4_^−^] is one of the prominent crystalline systems in which the ClO_4_^−^ anions are spatially arranged to form a one-dimensional wire-like feature in the extended lattice, Figure 2a, with *r* = 3.483 Å, and ∠Cl–O···Cl and ∠O···Cl–O = 175.7°. However, in the crystal structure of [CH_4_N_5_^+^, CH_3_N_5_, ClO_4_^−^], Figure 2b, the corresponding geometry has *r* = 3.936 Å and ∠O···Cl–O = 153.5°, indicating variability in the intermolecular arrangement driven by the nature and size of the cation.

The ClO_4_^−^ anions in the crystal structure of [C_10_H_12_Ag_2_N_10_^4+^, 4(ClO_4_^−^), 2(H_2_O)] are arranged such that the O···Cl distances are 3.805, 3.718, 3.856, and 3.850 Å, most of which are directional. If these contacts are considered genuine attractions, they would suggest the formation of a two-dimensional inorganic layer by the anions (Figure 2c). A similar geometric arrangement between the anions is observed in the crystal structure of [C_4_H_7_N_4_O^+^, ClO_4_^−^] (CSD ref: ENISED). In the crystal structure of [C_4_H_6_N_5_O_2_^+^, ClO_4_^−^] (CSD ref: ONAYAH), the intermolecular distances are 3.718 and 3.997 Å, which are comparable to those observed above, as well as to those in the crystal structure of [(C_2_H_8_AgN_2_^+^)_n_, (ClO_4_^−^)_n_] (CSD ref: AGENPC11).

The connectivity between IO_4_^−^ anions in the crystal structure of [C_6_H_5_N_2_^+^, IO_4_^−^], forming an overall two-dimensional network governed by directional interactions despite inequivalent O···I separations, is illustrated in Figure 2d. One contact type is notably shorter and highly directional (*r* = 3.330 Å, ∠O–I···O = 175.9°). The other two contact types are significantly longer (*r* = 3.940 and 3.952 Å) and exhibit mixed angular features; for example, one is associated with ∠I–O···O = 170.1° and ∠O···I–O = 160.5°. This combination of competing angular preferences makes it difficult to unambiguously designate the interaction as either oxygen-centered or iodine-centered. While only O···I close contacts are shown, O···O contacts between the anions cannot be overlooked.

The Rb^+^ cation is locally octahedral in [C_6_H_14_N_2_^2+^, 3(IO_4_^−^), Rb^+^], being surrounded by six anions that form a cage-like structure enclosing the cation, with six Rb···I distances of 3.727 Å (Figure 2e, left). The junction region between a pair of O atoms in each IO_4_^−^ interacts with the cation, forming pairs of Rb···O close contacts (*r* = 3.048 and 3.107 Å). Both correspond to alkaline bonding, with Rb acting as the electrophile. Similarly, the organic cation is enclosed within a cage formed by 12 IO_4_^−^ units (Figure 2e, middle), where each anion forms at least three O···I close contacts with neighboring anions, with *r* (∠O–I···O) values of 3.527 Å (173.0°) and 3.425 Å (176.7°). This indicates that the overall three-dimensional crystal structure arises from these interactions, along with multiple hydrogen bonds, which collectively stabilize the ordered lattice (Figure 2e, last). A very similar bonding feature is observed in the crystal structure of molecular perovskites [C_6_H_14_N_2_^2+^, 3(IO_4_^−^), NH_4_^+^] (CSD ref: TEJBIX01) [27], in which both NH_4_^+^ and C_6_H_14_N_2_^2+^ are enclosed within cages formed by six and twelve IO_4_^−^ units, respectively.

## 4. Computational Chemistry of Organic-Inorganic Crystal Systems

### 4.1. Case I

**Figure 3:**
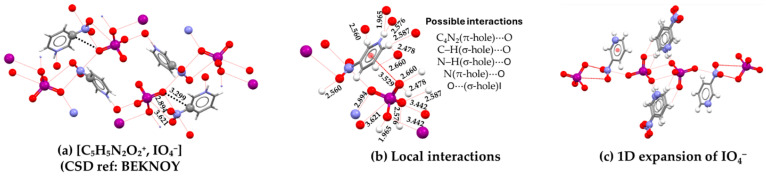
(**a**) Unit cell of the [C_5_H_5_N_2_O_2_^+^·IO_4_^−^] crystal structure, highlighting selected N···O, C···O, and O···I close contacts corresponding to pnictogen-, tetrel-, and chalcogen-bonding interactions, respectively. (**b**) Local nature of the intermolecular bonding interactions between the cation and anion. (**c**) Expanded view of the linkage within and beyond the (IO_4_^−^)_2_ dimer and the hydrogen bonds that hold the anions together. Selected bond distances and angles are in Å and °, respectively. Dashed lines represent close contacts.

The assembly between the organic cation and the inorganic anion in the crystal structure of [C_5_H_5_N_2_O_2_^+^, IO_4_^−^] (CSD ref: BEKNOY) [13], Figure 3a, comprises N···O (*r* = 2.894 Å, ∠C–N···O = 92.5°), C···O (*r* = 3.299 Å, ∠C–C···O = 114.7°), and O···I (*r* = 3.621 Å, ∠N=O···I = 91.2° ∠O–I···O = 162.3°) close contacts. These attractive interactions are inferred from the intermolecular distances, and directional characteristics, together with the overall electrophilic nature of the cation.

Apart from those above, several additional attractive engagements are also feasible. These include the C_4_N_2_(π-hole)···O, C–H(σ-hole)···O, and N–H(σ-hole)···O contacts (Figure 3b), which collectively contribute to the overall stability of the crystal framework.

Many of the H-assisted close contacts are weak, with H···O distances in the range 2.4–2.7 Å; nevertheless, they are all shorter than the sum of the van der Waals radii of H and O (2.75 Å; O = 1.55 Å and H = 1.20 Å) and therefore cannot be disregarded as hydrogen bonds. The strongest hydrogen bond involves the protonated H donor, which forms a significantly shorter interaction (*r* = 1.965 Å, ∠N–H···O = 142.6°). The middle portion of Figure 3c further shows that the close dimeric arrangement between the IO_4_^−^ anions is a forced consequence of the network of hydrogen bonds provided by several surrounding organic cations.

Although the O···I interaction between the O in the –NO_2_ fragment of the cation and anion’s I site is (negative) σ-hole-centered, it is likely a secondary, geometry-driven feature that arises as a consequence of the primary interactions, particularly the O···H and N···O contacts. As shown in Figure 3b,c, the hydrogen atom of both the N–H and C–H fragments of the organic cation bridge the anions in a competitive environment, thereby polarizing them and locally promoting the formation of (IO_4_^−^)_2_ dimers.

The O···I interaction between the cation and anion can be described as a chalcogen bond owing to the electrophilic nature of the cation, whereas the corresponding interaction between the anions (*r* = 3.442 Å, ∠O–I···O = 177.0°) may or may not be regarded as halogen bonding. The assignment of the latter relies on the assumption that the anion may or may not be sufficiently polarized by the extensive hydrogen-bonding network provided by the cation. This issue is clarified below, which delineates the role of this network and shows that it cannot be fully captured by any dimer model.

**Figure 4:**
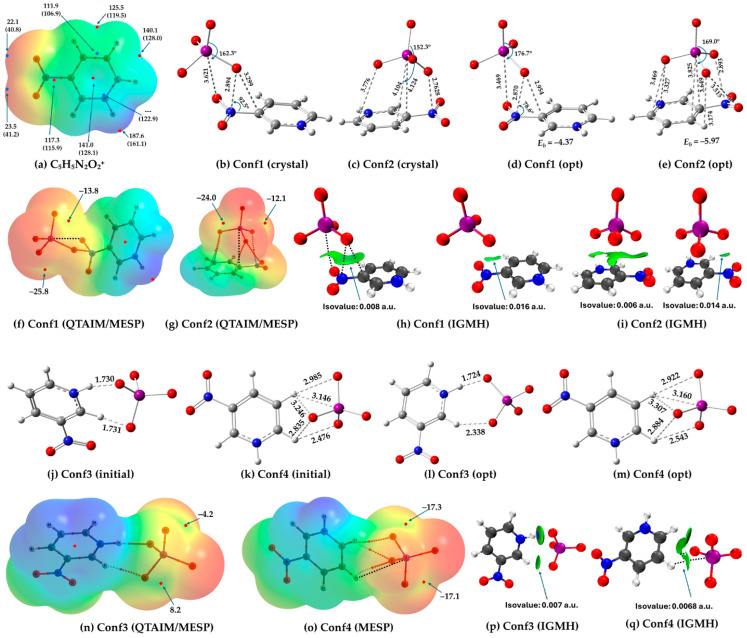
(**a**) QTAIM molecular graph superimposed on the MESP plot of the C_5_H_5_N_2_O_2_^+^ organic cation, mapped on the 0.0013 a.u. electron density isosurface (values in parentheses correspond to the gas phase, with the potential mapped on the 0.001 a.u. electron density isosurface). (**b**,**c**) C_5_H_5_N_2_O_2_^+^···IO_4_^−^ dimeric configurations Conf1 and Conf2 extracted from the crystal geometry. (**d**,**e**) Corresponding energy-minimized geometries. (**f**,**g**) QTAIM molecular graphs superimposed on the MESP plots of the optimized dimers, with the potential mapped on the 0.001 a.u. isodensity envelope. (**h**,**i**) IGMH isosurface plots of the corresponding complexes. (**j**–**q**) Analogous features for Conf3 and Conf4; in these cases, the H atoms in the rings of the initial structures were rebuilt due to their lower accuracy in the crystal geometry. Close contacts are indicated by dotted lines, and selected distances and angles are given in Å and degrees, respectively. Electrostatic potentials are in kcal mol^−1^. The color scale is such that red denotes the most negative regions and blue denotes the most positive regions. The black dotted lines missing in the molecular graphs in (**f**,**g**,**o**) were manually added to indicate attractive interactions.

The assignment above is consistent with the MESP results of the organic cation shown in Figure 4a. In the gas phase, the electrostatic potential mapped on the 0.001 a.u. isodensity envelope reveals a clear π-hole above and below the C–N(O_2_) bond (*V_S,max_* = 117.3 kcal mol^−1^), along with a pair of equivalent minima above and below the arene C=C bond (*V_S,min_* = 111.9 kcal mol^−1^). The entire molecular surface remains positive, including the π-hole at the centroid of the C_5_N ring (*V_S,max_* = 141.0 kcal mol^−1^). In solution, a 0.0013 a.u. isodensity envelope was required, as the π-hole features between the C and N atoms of the C–N(O_2_) fragment were not visible when the potential was mapped on the 0.001 a.u. isodensity envelope, indicating attenuation of these intrinsic features upon solvation.

The geometry of the dimer, extracted from the crystal structure (Figure 4b,c), and their energy-minimized counterparts (Figure 4d,e) show that the monomers do not reorganize significantly upon optimization. We refer to the two dimers as Conf1 and Conf2; they have binding energies of −4.37 and −5.97 kcal mol^−1^, respectively.

QTAIM molecular graphs superimposed with MESP plots reveal only a single N···O interaction between the cation and anion in Conf1 (Figure 4f), whereas multiple interactions are observed in Conf2, including (O_2_)N···O, (C=N)···O, C···O, and (NO)O···O, all of which are coulombic in nature (Figure 4g). Interestingly, QTAIM does not identify close contacts such as O···I in Conf1 or C···I in Conf2. The directional interaction centered on I’s negative σ-hole, observed in both configurations on geometric grounds, is better described as a chalcogen bond in Conf1 and a tetrel bond in Conf2, rather than a halogen bond (Figure 4f). Our rationalization is further supported by the IGM analysis discussed below.

The O···O contact in Conf2 (3.515 Å) is relatively long compared with twice the van der Waals radius of oxygen (3.10 Å), suggesting that this interaction is extremely weak. The (C=C)π···O contacts, with C···O distances of 3.649 and 3.825 Å, correspond to tetrel-bonding interactions and do not represent chalcogen bonding. In the crystal structure, a similar interaction is observed but is even weaker, given the longer intermolecular separation of approximately 4.1 Å (Figure 4c). The N···O close contact corresponds to a pnictogen bond and is one of the primary interactions in the crystal. The C···O distance of 3.776 Å marked in the crystal geometry in Figure 4c represents a π-hole-centered (C_5_N)···O interaction.

The hyperconjugative interactions in Conf2 involve LP(3) O → BD*(2) (C=N) and LP(3) O → BD*(2) (C=C), with *E*^(2)^ values of 0.51 and 0.63 kcal mol^−1^, respectively, where LP and BD denote lone-pair and bonding orbitals, respectively. The C···I interaction is described by σ(I–O) → RY*(9) C, with *E*^(2)^ values in the range 0.10–0.17 kcal mol^−1^, where each of the three O–I bonds oriented toward the ring is involved and RY represents a Rydberg-type orbital. This collective interaction indicates the presence of a tetrel bond.

For Conf1, LP(3)O → BD*(2)(C=O) is identified as the strongest delocalization interaction, with an *E*^(2)^ value of 1.04 kcal mol^−1^, even though the MESP model suggests an interaction between the maximum potential associated with the π-hole region near the center of the C–N(O_2_) fragment of the cation and a nucleophilic O atom of IO_4_^−^ oriented toward it. The σ(I–O) → RY*(8)C and LP(2)O → σ*(I–O) interactions (*E*^(2)^ values of 0.06 and 0.19 kcal mol^−1^, respectively) may be indicative of possible tetrel and chalcogen bonding interactions, respectively.

The electrostatic potential on the surface of iodine in either of the two configurations of the C_5_H_5_N_2_O_2_^+^···IO_4_^−^ complex is not positive. Each iodine atom hosts three negative σ-holes along the extensions of three O–I bonds, with *V_S,max_* values of −35.9 (−24.0), −25.9 (−12.1), and −13.8 (−7.1) kcal mol^−1^, indicating that these cannot act as electrophiles to form halogen bonds with nearby negative sites; the values in parentheses correspond to the strongly bound complex. The fourth σ-hole on the same atom, which was found in the isolated molecular entity, is effectively annihilated upon interaction with the oxygen atom of the –NO_2_ fragment (or with the carbon framework in Conf2) of the cation. Moreover, the global maximum of the electrostatic potential is located at the protonated site of the cation, with a *V_S,max_* value of 133.5 (122.1) kcal mol^−1^ on hydrogen along the N–H bond extension, whereas all minima are located on the oxygen atoms of the anion, with *V_S,min_* values of −70.6 (−61.4), −74.6 (−61.3), and −79.5 (−68.0) kcal mol^−1^. Although this description applies to the two specific configurations considered here, alternative configurations may yield a slightly different electrostatic picture.

The IGMH isosurface volume disappears only at an isovalue of 0.018 a.u. for Conf1. At slightly lower isovalues (0.017 and 0.016 a.u.), faint isosurface features remain (Figure 4h). This corresponds to an interaction between the (N–C)_π_ fragment associated with –NO_2_ of the cation and an oxygen atom of the anion. It is not a purely tetrel or purely pnictogen bond, as it occurs in the bonding region of the C=N fragment and involves a nucleophilic oxygen atom, reflecting a mixed and highly delocalized interaction. The O···I interaction appears only at lower isovalues around 0.008 a.u., indicating that it is relatively weak.

For Conf2, the IGMH isosurface remains pronounced even at an isovalue of 0.010 a.u., and at a lower value of 0.006 a.u. all types of interactions persist, including C···I, C–H···O, and (C=N)_π_···O, and (C–N)_π_···O contacts (Figure 4i). Among these, the dominant interaction is not I···C but (C–N)_π_···O, whereas the strip-like green isosurface corresponds to the C–H···O close contact. At an isovalue of 0.014 a.u., only the N_π_···O interaction remains, indicating that the remaining contacts are secondary in nature. We did not observe any isosurface volume corresponding to an O···O close contact, indicating that the interaction identified by QTAIM is likely spurious.

The initial geometries of Conf3 and Conf4, constructed to closely resemble those observed in the extended crystal structure, considering that the H-atom positions in the experimental structures were less reliable, as shown in Figure 4j,k. The corresponding energy-minimized geometries are shown in Figure 4l,m, which reveal only minor rearrangement between the monomers upon optimization.

QTAIM’s molecular graph in Figure 4n shows Conf3 exhibits two hydrogen-bonding interactions: one N–H···O contact (*r* = 1.724 Å, ∠N–H···O = 170.9°) and one C–H···O contact (*r* = 2.338 Å, ∠C–H···O = 150.9°). NBO analysis indicates that the N–H···O interaction is dominated by LP(1)O → σ*(N–H) and LP(3)O → σ*(N–H) donor–acceptor interactions with corresponding *E*^(2)^ stabilization energies of 6.79 and 23.48 kcal mol^−1^, respectively. Similarly, the C–H···O interaction is described by LP(1)O → σ*(C–H), LP(2)O → σ*(C–H), and LP(3)O → σ*(C–H) interactions with *E*^(2)^ values of 0.69, 0.10, and 2.20 kcal mol^−1^, respectively.

Conf4 in Figure 4m displays a C–H···I close contact with *r* = 3.160 Å, ∠C–H···I = 119.4°, and ∠O–I···H = 171.6°. This interaction is directional with respect to the negative σ-hole on iodine and is best described as a hydrogen bond with iodine acting as a Lewis base. It is accompanied by two additional H···O close contacts involving the same proton, with *r* = 2.922 Å (∠C–H···O = 143.0°) and 2.954 Å (∠C–H···O = 114.1°), indicating that this H atom behaves as a trifurcated proton donor center. Similar C–H···O contacts are also identified for a neighboring C–H group interacting with the anion oxygen atoms, with H···O distances of 2.543 and 2.884 Å. The NBO analysis predicts LP(2)/LP(3) O → σ*(C–H) hyperconjugative interactions with *E*^(2)^ values of 0.13/0.73 and 0.06/0.37 kcal mol^−1^ for the respective contacts. By contrast, the O···I interaction is described by σ(O–I) → RY*(3) with an *E*^(2)^ value of 0.10 kcal mol^−1^. The second-order analysis also predicts an LP(3) O → BD*(2) (C=N) interaction associated with the C_5_N ring with an *E*^(2)^ of 0.90 kcal mol^−1^; however, this donor–acceptor interaction is not supported by the intermolecular geometry, as the O atom is not oriented toward the C=N bond and no corresponding intermolecular contact is identified.

While QTAIM fails to reveal the C–H···I hydrogen bond in Conf4 (Figure 4o), it captures most of the hydrogen bonds both in Conf3 and Conf4. The bluish-green isosurface between N–H and O in Conf3 in Figure 4p indicates a comparatively stronger interaction than the other contacts present in Conf3 and Conf4. The IGMH isosurface confirms a weak but continuous interaction region spanning this trifurcated donor environment (Figure 4q).

The three σ-holes on iodine in IO_4_^−^ exhibit different degrees of polarization in Conf3 and Conf4 upon interaction with the cation. In Conf3, one σ-hole becomes positive (*V_S,max_* = 8.1 kcal mol^−1^), whereas the other two remain negative (−4.2 and −11.5 kcal mol^−1^), indicating partial sign inversion induced by the cation (two of them shown in Figure 4n). In Conf4, all three σ-holes on iodine remain negative (−1.1, −17.1, and −17.3 kcal mol^−1^), although they are noticeably polarized relative to the isolated anion (two shown in Figure 4o).

The binding energies across the dimer series follow the stability trend: Conf3 (−6.06) > Conf1 (−5.97) > Conf2 (−4.37) > Conf4 (−3.38 kcal mol^−1^), indicating that the hydrogen-bonded environment involving the protonated amine leads to the most stable complex.

### 4.2. Case II

**Figure 5:**
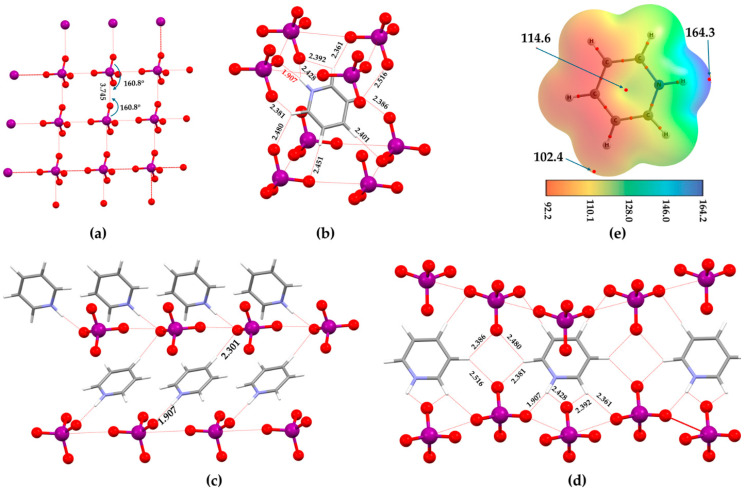
The nature of the special arrangement between (**a**) inorganic anions and (**b**–**d**) organic–inorganic anions in the crystal structure of pyridinium periodate (CSD ref: HOHMOG05). (**e**) QTAIM molecular graph overlaid with the MESP plot, showing the maxima of the potential as tiny red circles (values in kcal mol^−1^). Selected bond distances and angles are given in Å and degrees, respectively. Intermolecular interactions are highlighted as dotted lines in red.

Pyridinium periodate, [C_5_H_6_N^+^, IO_4_^−^] (CSD ref: HOHMOG05), is an interesting system featuring O···I contacts in two crystallographic directions, thereby forming a two-dimensional inorganic sheet, as the iodine atom participates in a pair of O···I contacts along orthogonal directions. The O–I···O and I–O···I angles are nearly equivalent (Figure 5a). The back-to-back arrangement of the anions suggests directional σ-hole-centered interactions when the MESP surface is considered; however, the corresponding σ-holes on the isolated anion are negative.

Figure 5b shows that the dominant interactions driving self-assembly are hydrogen bonds. The cation participates by forming multiple N–H···O and C–H···O contacts with surrounding periodate anions, bringing them into close proximity and enforcing the observed anion–anion arrangement driven by O···I close contacts. Most of these H···O distances fall in the range 2.3–2.6 Å. The strongest contact involves the protonated hydrogen atom, with an H···O distance of 1.907 Å and an N–H···O angle of 163.3°, similar to that observed in the related crystal, C_5_H_5_N_2_O_2_^+^···IO_4_^−^ (CSD ref: BEKNOY). The local hydrogen-bonding network can be seen in Figure 5c,d, where the organic cations connect and stabilize the layers.

To provide further insight into the nature of intermolecular interactions, as suggested by the crystal geometries, we extracted several dimer models from the crystal structures. Energy minimization indicates that the monomers in these models do not undergo significant reorganization, but they adjust slightly to maximize intermolecular interactions, consistent with observations in similar systems (see Cases I above and III-V below).

**Figure 6:**
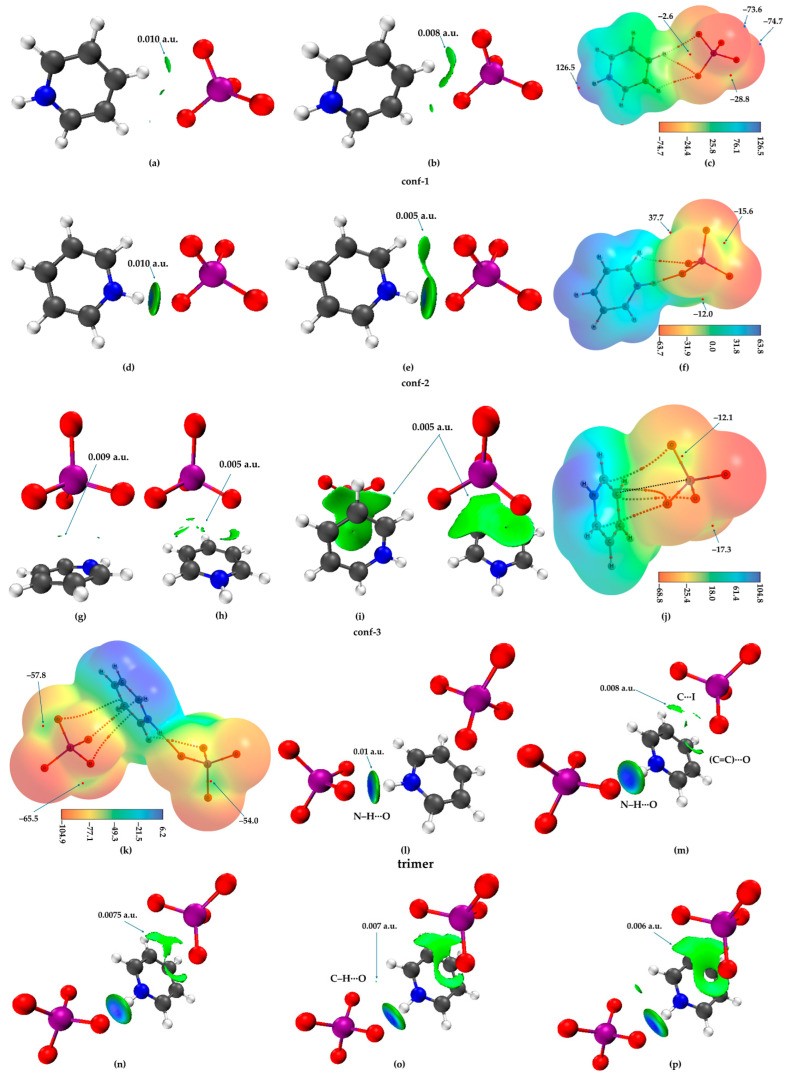
(**a**,**b**) IGMH-based isosurface volumes at two different isovalues; (**c**) QTAIM-superimposed MESP plot of Conf1 obtained on the SMD geometry of C_5_H_6_N^+^⋯IO_4_^−^; (**d**–**f**) and (**g**–**j**) corresponding plots for Conf2 and Conf3, respectively; (**k**) QTAIM-superimposed MESP plot of C_5_H_6_N^+^⋯(IO_4_^−^)_2_; and (**l**–**p**) IGMH-based isosurface volumes at five different isovalues. The maxima of the electrostatic potential (small red circles indicated by arrows) are given in kcal mol^−1^, whereas bond paths and critical points are shown as solid lines (in atom colors) and red dots along the bond paths, respectively. The black dotted line in (**j**) is manually constructed to indicate the C···I interaction.

Figure 6a,b shows IGMH isosurface plots of Conf-1 optimized in solution. The dominant C–H⋯O hydrogen bond (*r* = 2.507 Å, C–H⋯O = 172.3°) is visible at an isovalue of 0.010 a.u. Two additional interactions—one between another adjacent O site and the same C–H group (*r* = 2.715 Å, C–H⋯O = 121.0°) and the other involving the nearest C–H fragment of the cation (*r* = 2.663 Å, C–H⋯O = 122.3°)—are weaker at this isovalue but become more pronounced when the isovalue is lowered to 0.008 a.u. (Figure 6b). Among these, the latter C–H⋯O contact is the weakest, consistent with its slightly longer bond distance. The second-order NBO analysis shows LP(3)O → σ*(C–H) type delocalization, which accounts for the three interactions described above, with stabilization energies *E*^(2)^ values of 1.60, 0.38, and 0.14 kcal mol^−1^.

The assembly of the ion pairs leading to the complex does not significantly polarize the IO_4_^−^ anion to induce positive sites, as all four σ-holes on its electrostatic surface remain negative (*V_S,max_* = −2.6, −12.1, −28.8, and −31.8 kcal mol^−1^; two shown in Figure 6c). This demonstrates that the anion continues to act solely as a nucleophile and cannot function as a halogen-bond donor in this specific configuration. The strongest σ-hole is located on the protonated amine (*V_S,max_* = 126.5 kcal mol^−1^), whereas the most nucleophilic sites are distributed on the O atoms of IO_4_^−^ (*V_S,min_* = −73.6 and −74.7 kcal mol^−1^).

For Conf-2, shown in Figure 6d, the prominent chemical interaction is the N–H⋯O hydrogen bond (*r* = 1.798 Å, N–H⋯O = 171.1°), as observed in the crystal structure (see above), and no other interactions were revealed by the IGMH analysis when an isovalue of 0.010 a.u. was used. This N–H⋯O interaction disappears only at an isovalue of 0.062 a.u., whereas the C–H⋯O interaction becomes visible at 0.0075 a.u. (Figure 6e), associated with *r* = 2.899 Å, C–H⋯O = 112.6°. Quantitatively, the C–H⋯O interaction is approximately 12% weaker than the N–H⋯O hydrogen bond, when the two isovalues above are compared. Clearly, the binding energy of the complex (*E_b_* = −5.29 kcal mol^−1^) is largely due to this latter interaction.

The MESP plot of the complex shows three σ-holes on the surface of iodine in IO_4_^−^ instead of four: two are negative (*V_S,max_* = −12.0 and −15.6 kcal mol^−1^) and one is positive (*V_S,max_* = 36.7 kcal mol^−1^); two of them are shown in Figure 6f. The O sites in IO_4_^−^ remain negative, while the organic cation is entirely positive. The apparent neutralization of a σ-hole on I observed at the 0.001 a.u. MESP surface is sensitive to the choice of electron density isosurface: when a slightly higher isovalue of 0.0013 a.u. is used, the previously missing σ-hole appears positive, indicating that the magnitude and sign of σ-holes depend on the electron density surface chosen for MESP evaluation. The strengths of all four σ-holes on I in IO_4_^−^ are as follows: two negative (*V_S,max_* = −7.4 and −10.4 kcal mol^−1^) and two positive (*V_S,max_* = 40.7 and 16.3 kcal mol^−1^), suggesting that the anion in the complex can act both as a halogen bond donor and acceptor when in proximity to a nucleophile on another interacting species.

The geometric stability of Conf-3 arises from a multi-bonding environment composed of (C_5_N)_π-hole_···O, C–H···O, and O···I close contacts. The latter is a highly directional, C-centered negative I’s σ-hole-driven interaction (*r* = 3.724 Å, ∠C···I–O = 84.9°, ∠C···I–O = 176.7°), whereas the former two are comparatively non-linear; for C–H···O, *r* = 3.180 (3.477) Å and ∠C–H···O = 90.6 (83.2°). QTAIM does not recognize either of these interactions; instead of the hydrogen bonds, it predicts a pair of C···O close contacts (*r* = 3.518 (3.308) Å and ∠C···O–I = 94.3° (125.9°)). The (C_5_N)_π-hole_···O interaction is characterized by a centroid-to-O distance of 3.182 Å, corresponding to an orthogonal arrangement. QTAIM also identifies two additional C···O close contacts that effectively represent this π-hole interaction. The geometry based assignment is consistent with the nature of the IGMH isosurface volumes between the interacting molecular entities, which are nearly absent at an isovalue of 0.009 a.u. (Figure 6g) and become evident only at isovalues lower than 0.008 a.u. (Figure 6h,i), suggesting that the O···I chalcogen bond is weaker than the other two interaction types.

The three non-interacting σ-holes on iodine in IO_4_^−^ in the complex are negative, with *V_S,max_* values of −12.1, −17.3, and −21.9 kcal mol^−1^ (two shown in Figure 6j), implying that they may act as halogen-bond acceptors. The global maximum of the electrostatic potential is located at the protonated H atom (*V_S,max_* = 104.8 kcal mol^−1^), whereas the global minimum (*V_S,min_* = −68.8 kcal mol^−1^) resides at a non-interacting O site, suggesting that anionic and cationic s remain nucleophilic and electrophilic in the complex, respectively.

Among the three ion pairs examined, the *E_b_* follows this order: Conf-2 (−5.29 kcal mol^−1^) > Conf-3 (−4.40 kcal mol^−1^) > Conf-1 (−1.90 kcal mol^−1^). The larger stabilization of Conf-2 is likely associated with a larger elongation of the aminated N–H bond (*r* = 1.032 Å) in the complex relative to the isolated value of 1.016 Å, corresponding to an increase of approximately 1.6%. By contrast, the C–H bond involved in the dominant C–H⋯O interaction elongates from 1.0789 Å in the isolated monomer to 1.0823 Å in Conf-1, corresponding to an increase of approximately 0.32%.

We have also examined a trimer, C_5_H_5_N_2_O_2_^+^···(IO_4_^−^)_2_ (Figure 6k), which consists of two anion units and one cation unit in a 1:2 arrangement. Although this complex has an overall charge of −1, the MESP analysis shows that the C_5_H_5_N_2_O_2_^+^···(IO_4_^−^)_2_ system is not entirely negative. σ-holes along several C–H extensions are positive (*V_S,max_* values of 4.9, 5.0, and 6.3 kcal mol^−1^), whereas the IO_4_^−^ monomers are entirely negative, exhibiting negative σ-holes along the O–I bond extensions (−65.5, −61.3, −57.8, −53.0, and −54.0 kcal mol^−1^; three of them are shown in Figure 6k). The strongly negative nature of the σ-holes on iodine indicates that it cannot act as a halogen-bond donor. Accordingly, the C···I close contact discussed below is not a halogen bond but is better described as a tetrel bond.

Based on the energy-minimized geometry, the dominant intermolecular interaction within the supermolecular assembly is an N–H···O hydrogen bond (*r* = 1.798 and ∠N–H···O = 177.7°), accompanied by a C···I close contact (*r* = 3.713 Å, ∠C=C···I = 87.5°, ∠C···I–O = 176.8°) and (C=C)···O interactions in the range 3.32–3.36 Å. A possible C–H···O interaction may also be feasible, even though the H···O separation (2.872 Å) is slightly longer than the sum of the van der Waals radii (2.75 Å; O = 1.55 Å and H = 1.20 Å). These interactions are supported by the QTAIM topology, although QTAIM fails to reveal the C···I close contact.

The geometry-based interpretation given above is consistent with the IGMH analysis (Figure 6l–p), which shows that the N–H···O interaction is the strongest, visible even at an isovalue of 0.01 a.u. (Figure 6l). The C···I interaction is weaker, appearing only as a faint isosurface at an isovalue of 0.008 a.u. (Figure 6m). A π-hole···O interaction is also observed, faint at 0.008 a.u. (Figure 6m,n) but more pronounced at 0.006 a.u. (Figure 6p). Additional tertiary interactions, including the C–H···O contact, are not visible at 0.0075 a.u. (Figure 6n) but appear at 0.007 a.u. and become more diffuse at 0.006 a.u. (Figure 6p), indicating their comparatively weak nature.

The overall binding energy of C_5_H_5_N_2_O_2_^+^···(IO_4_^−^)_2_ is −8.99 kcal mol^−1^. Using the most stable C_5_H_5_N_2_O_2_^+^···IO_4_^−^ dimer as reference, the stepwise binding energy for addition of the second anion is −3.70 kcal mol^−1^, which is smaller than the first binding event, indicating negative cooperativity. This reduction arises from the involvement of different interaction sites in the trimer compared with those operative in the dimer.

### 4.3. Case III

What are the possible intermolecular interactions in the ion-pair crystal lattice of 3-carboxypyridinium periodate, [C_6_H_6_NO_2_^+^, IO_4_^−^] (CSD ref: BEKNUE)?

**Figure 7:**
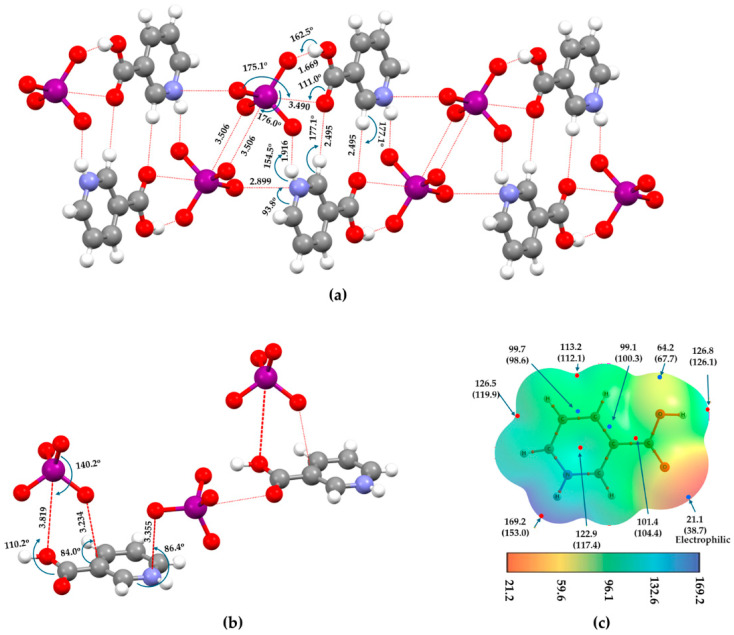
(**a**,**b**) Illustration of possible close contacts between interacting monomers in the crystal structure of 3-carboxypyridinium periodate (CSD ref: BEKNUE). (**c**) QTAIM molecular graph at the M06-2X/SMD level, superimposed on the MESP plot of the organic cation, showing the various minima (tiny blue circles) and maxima (tiny red circles) of the electrostatic potential (kcal mol^−1^); values in parentheses correspond to the gas-phase results. Selected bond distances and bond angles are given in Å and degrees, respectively.

Figure 7a shows that several intermolecular contacts are apparent between the monomers in the crystal. Among them are an (O)H···O interaction and an O···I contact. The O and H atoms of the –OH group of the C_6_H_6_NO_2_^+^ cation act simultaneously as an electrophilic donor for the development of two noncovalent interactions. This donates a hydrogen atom to the nearest oxygen of one IO_4_^−^ moiety, forming the strongest O–H···O contact (*r* = 1.669 Å, O–H···O = 162.5°), and its oxygen atom interacts with the iodine of another IO_4_^−^ anion (*r* = 3.819 Å, C–O···I = 110.2°), which is a chalcogen bond. The former was assigned as a hydrogen bond [13], whereas the possibility of the latter was assigned as a halogen bond. Similarly, our geometry-based analysis suggests that the C···O and N···O close contacts shown in Figure 7b correspond to tetrel and pnictogen bonds, respectively.

The directional feature O···(σ-hole)I observed between the interacting monomers is insufficient to label the interaction as halogen bonding. As shown in Figure 7a, the H···O hydrogen bond appears to be the primary interaction governing the ion-pair geometry, whereas the O···I contact likely arises as a secondary interaction, as reflected in its longer bond distance. It should be emphasized that the interacting oxygen atoms in the –COOH group do not act as a nucleophilic partner, since it is an integral part of the cation and its electrostatic environment is strongly influenced by the surrounding hydrogen-bond network. Therefore, the O···I contact is better described as a weak, secondary chalcogen-bonding interaction.

However, the O···I close contact was interpreted as a halogen bond based on its directionality and a proposed charge transfer from the lone pair of the carboxylic oxygen to the σ* antibonding orbital of the I–O bond [13]. Such an interpretation is questionable, because charge-transfer stabilization alone does not establish halogen bonding when the interacting halogen lacks an electrophilic site and when the partner oxygen is not nucleophilic in electrostatic terms.

To further substantiate the above point, we examine the electrostatic potential of the cation (Figure 7c). The surface is positive throughout, whether at the centroid of the arene moiety, along the N–H and C–H bond extensions, or at the oxygen lone-pair regions. Under such conditions, what should be expected when the oxygen site of this molecule engages in a noncovalent interaction with the negative σ-hole on the iodine atom of the anion? Should the resulting I···O contact be classified as a halogen bond or a chalcogen bond? It is clearly the latter, not the former, since both the oxygen atoms in the –COOH group of the cation act as the electrophilic site, despite the presence of a minimum of potential (Figure 7c). This view is not counterintuitive; rather, it follows the classical coulombic picture used to recognize halogen bonding and chalcogen bonding interactions.

A related question concerns the pair of intermolecular H···O contacts between the cations (*r* = 2.495 Å, C–H···O = 177.1°), as shown in Figure 7a. These interactions are weak and appear to primarily play a structural role. Unlike the N–H···O hydrogen bonds formed between the protonated amine group (–N–H) of the organic cation and an oxygen atom of a nearby IO_4_^−^ anion—which are classical, strong hydrogen bonds—the C–H···O contacts between the cations are better described as secondary, geometry-dictated contacts, arising as a consequence of the primary acid-base coulombic attractions between the organic cation and the inorganic anions.

**Figure 8:**
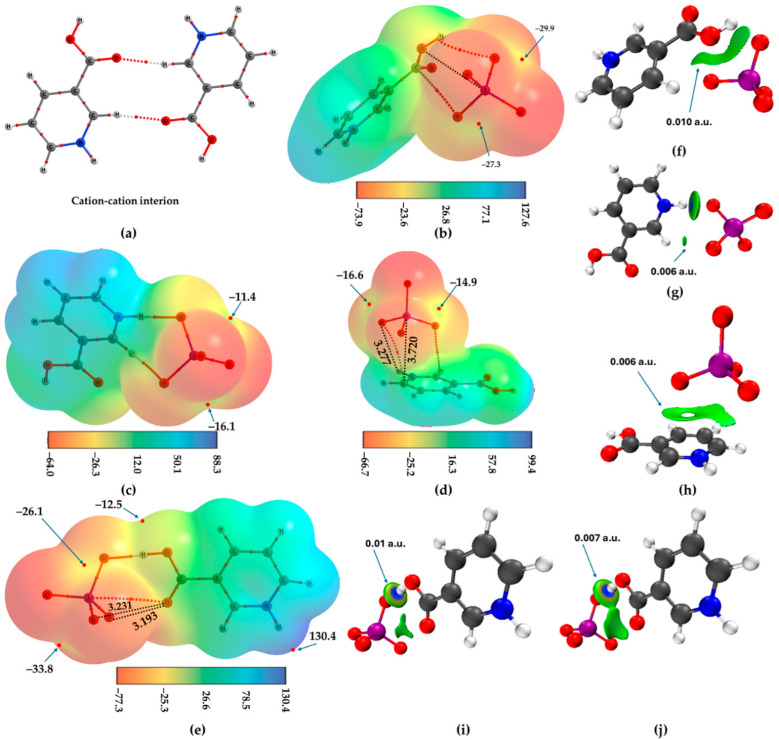
(**a**) QTAIM molecular graph of the [C_6_H_6_NO_2_^+^]_2_ dimer, showing bond paths as solid and dotted lines in atom-specific colors, and bond critical points as small red spheres along the bond paths. (**b**–**e**) QTAIM molecular graphs superimposed with MESP plots for four C_6_H_6_NO_2_^+^···IO_4_^−^ dimers. (**f**–**j**) IGMH-based isosurface plots of the corresponding dimers. Selected bond distances in (**d**–**e**) are given in Å. The black dotted lines in (**b**,**d**,**e**) are manually constructed to indicate possible close contacts not identified by QTAIM. Electrostatic potentials are in kcal mol^−1^.

To confirm this, we energy-minimized a pair of cations in the gas phase (Figure 8a), as observed in the crystal (Figure 7a). Interestingly, unlike the (IO_4_^−^)_2_ dimers, the monomers did not repel each other to infinite separation. The C–H···O close contacts (*r* = 2.202 Å, C–H···O = 169.5°) are slightly strengthened in the gas phase compared to those found in the crystal. Despite this geometric stabilization, the binding energy remains positive (*E_b_* = 31.03 kcal mol^−1^), indicating that each H···O contact contributes approximately 15.51 kcal mol^−1^ to the overall complex stability. The uncorrected and BSSE-corrected interaction energies for the complex are 30.51 and 30.60 kcal mol^−1^, respectively, computed using the optimized geometry of the complex, with the monomer energies evaluated on their complexed geometries. This result shows that the closeness between the cationic sites could apparently be influenced by coulombic interactions and is not strictly valid. The observed attraction arises from unequal charge densities and directional orbital effects, such that one site is more electron-depleted than the other, even though both regions are electrophilic, analogous to what was reported previously [35,51]. The SAPT0 level analysis suggests that electrostatics, exchange, induction and dispersion components contribute approximately 29.2, 5.0, −4.4 and −3.8 kcal mol^−1^ to the overall total interaction energy.

The nature of the intermolecular interactions in the [C_6_H_6_NO_2_^+^]_2_ dication in solution is largely consistent with those observed in the gas phase. However, the C–H···O close contacts (*r* = 2.275 Å, C–H···O = 173.3°) are slightly elongated relative to the gas-phase geometry and may be appreciably underestimated compared to that in the crystalline geometry of [C_6_H_6_N O_2_^+^·IO_4_^−^] [13]. Nevertheless, the binding energy of the dimer is −1.05 kcal mol^−1^, indicating that the monomers remain weakly bound.

A second-order NBO analysis performed on the gas phase geometry of the [C_6_H_6_N O_2_^+^]_2_ dimer reveals charge transfer from the O lone pair to the antibonding σ*(C–H) orbital of the partnering cation, with *E*^(2)^ = 2.34 kcal mol^−1^ for LP(1) O → σ*(C–H). A much smaller contribution also arises from the donation of the C–H bonding orbital into the antibonding σ*(C–H) orbital, with *E*^(2)^ = 0.20 kcal mol^−1^ for BD(1) C–H → σ*(C–H). These donor–acceptor interactions are consistent with the formation of the observed noncovalent contacts. This, together with the directional nature of the interactions, prevents us from classifying them as classical hydrogen bonds; instead, we refer to them as “hydrogen-centered anti-electrostatic interactions.”

Figure 8b–e shows the molecular graph-superimposed MESP plots of the four investigated model ion pairs. In Figure 8a, the anion is positioned above the –COOH group, forming three potential intermolecular interactions, with a binding energy of $E_{b}=-4.56$ kcal mol^−1^. These correspond to (O)H···O, (C)C···O, and (H)O···I close contacts, accompanied by the annihilation of a negative σ-hole on the iodine atom of IO_4_^−^. The remaining three iodine σ-holes that do not interact with the organic cation remain negative, with $V_{S,\max}$ values of −21.1, −27.3, and −29.9 kcal mol^−1^ (two of which are shown in Figure 8b).

The intermolecular distances and angles associated with the three contacts referenced above are *r* = 2.392 Å, ∠O–H···O = 123.8°; *r* = 3.013 Å, ∠C–C···O = 86.8°; and *r* = 3.364 Å, ∠C–O···I = 95.7°, and ∠O–I···O = 172.2°, corresponding, respectively, to a σ-centered hydrogen bond, a π-centered tetrel bond, and a *p*-type lone-pair lump-centered chalcogen bond. Notably, in this case, the negative σ-hole is directionally aligned with the electrophilic region of the OH group. The QTAIM molecular graph failed to display the O···I close contact (the dotted line in black in Figure 8a); however, this was clearly confirmed by IGMH analysis at an isovalue of 0.010 a.u. (Figure 8f).

The conformer shown in Figure 8c has an $E_{b}=-5.61$ kcal mol^−1^. The N–H and C–H fragments of the cation form N–H···O and C–H···O hydrogen bonds with the anion, with bond distances and angles of 1.765 Å (174.7°) and 2.907 Å (112.4°), respectively. Two maxima of electrostatic potential were found on the iodine atom in IO_4_^−^ (*V_S,max_* = −11.4 and −16.1 kcal·mol^−1^), while the remaining two were effectively neutralized and could not be observed, even when an isodensity envelope of 0.0016 a.u. was applied to map the potential. This suggests that the electron density around the iodine atom is significantly altered during its interaction with the cation at equilibrium. Second-order NBO analysis confirms the presence of hyperconjugative interactions, including LP(3) O → σ(N–H) and LP(3) O → σ(C–H), with *E*^(2)^ values of 20.59 and 0.10 kcal mol^−1^, respectively, indicating that the latter interaction is roughly 200 times weaker than the former.

In the stable conformer ($E_{b}=-4.22$ kcal mol^−1^) shown in Figure 8d, the anion is positioned above the C–H fragment adjacent to the protonated ammonium site of the organic cation. The C···I bond distance is 3.720 Å, with ∠N–C···I = 88.7° and ∠O–I···O = 175.8°. The quasi-linear nature of the negative σ-hole prevents the C···O contact from being classified as a halogen bond; instead, it should be considered a carbon-centered tetrel bond. Additionally, notable close contacts between (C≡N)···O and (C=C)···O (*r* < 3.4 Å) suggest that the oxygen atom of the anion is attracted to the electron-deficient regions around the ring centroid, indicating a potential π-hole interaction. While QTAIM fails to identify the C···I tetrel bond through its bond-path topology, the (C=C)···O interaction is indeed revealed.

Three σ-holes on the iodine atom in IO_4_^−^ remain, while the fourth is effectively annihilated during the engagement between the ion pairs, with *V_S,max_* values of −9.4, −14.9, and −16.6 kcal·mol^−1^. These results suggest that the covalently bonded iodine atom in IO_4_^−^ cannot be considered a halogen-bond donor, even within the complex. The intermolecular interactions inferred solely from the geometric features are confirmed by the IGMH isosurface analysis shown in Figure 8g,h, including the O···I chalcogen bond.

The conformer shown in Figure 8e is the most energetically favorable ($E_{b}=-6.91$ kcal mol^−1^) among all conformers examined, likely due to the H···O strong hydrogen bond formed between the OH group and the anion, with *r* = 1.651 Å and ∠O–H···O = 178.9°. Additionally, part of the stability can be attributed to an O···I and a pair of O···O close contacts, which are ubiquitous given the orientation of the C=O fragment of the –COOH group. The former is characterized by *r* = 3.329 Å, ∠O–I···O = 178.9°, and ∠C–O···I = 110.5°, whereas the latter involves *r* = 3.231 Å and ∠C=O···O = 136.1° (and *r* = 3.193 Å and ∠C=O···O = 106.5°). These interactions are described as chalcogen bonds, where the σ-hole is not a prerequisite, but rather due to the electrophilic nature of the covalently bonded oxygen in the C=O group.

The electrostatic potential of the entire IO_4_^−^ anion in the complex is predominantly nucleophilic, with negative *V_S,min_* values concentrated around the covalently bonded oxygen atoms. By contrast, the regions along the I–O bond extensions, associated with σ-holes, exhibit negative *V_S,max_* values (*V_S,max_* = −26.1, −32.4, and −33.8 kcal mol^−1^), indicating that the IO_4_^−^ entity in this specific configuration cannot function as a halogen-bond donor. This result does not support the previously proposed halogen-bond donor role of the IO_4_^−^ unit [13]. In fact, the earlier study suggests a predominantly electrophilic character of iodine in the given environment, although detailed electrostatic potential descriptors (e.g., *V_S,max_* values) were not reported.

The geometry-based analysis of intermolecular interactions in all four ion pairs is supported by the IGMH isosurfaces in Figure 8f–j, with finer features visible at lower isovalues (~0.006 a.u.), while the most prominent interactions are best resolved at 0.01 a.u.

### 4.4. Case IV

**Figure 9:**
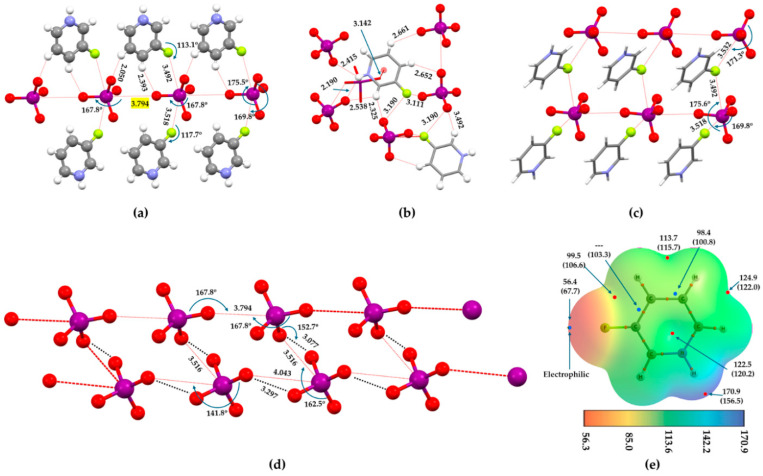
(**a**–**d**) Illustration of possible intermolecular close contacts (black and red dotted lines) in the crystal structure of 3-fluoropyridinium periodate [C_5_H_5_FN^+^·IO_4_^−^] (CSD ref: BEKPAM), identified based on bond distances (Å) and bond angles (degrees). (**e**) QTAIM molecular graph at the M06-2X/SMD level superimposed on the MESP plot of the organic cation (0.001 a.u. isodensity envelope used to map the potential); values of the electrostatic potential (kcal mol^−1^) given in parentheses correspond to the gas-phase results, and the “---” indicates that no minimum was observed on the SMD geometry.

3-Fluoropyridinium periodate (CSD refcode: BEKPAM) is another interesting system stabilized by an extensive network of hydrogen bonds, among other non-negligible close contacts, as shown in Figure 9a–d. The periodate anions are arranged back-to-back through O···I close contacts (see Figure 9a,c,d), while the fluorine atoms of the organic cation approach iodine along the extensions of the O–I bonds, forming directional inequivalent F···I contacts (see Figure 9a,c). At the same time, fluorine also interacts with oxygen sites, forming non-linear F···O close contacts (*r* = 3.1–3.2 Å, ∠C–F···O = 99.0–103.0°), as shown in Figure 9b. The possibility of O···O close contacts between the IO_4_^−^ anions should not be overlooked, as some are directional and lie within the sum of the van der Waals radii of oxygen (3.1 Å) (see black dotted lines in Figure 9d).

The I···O close contacts occur with different strengths, with separations of 3.516 Å in one direction and 3.789 Å in another, as shown in Figure 9d. Whether the latter, and especially the longer contact with *r* = 4.043 Å, can be regarded as a meaningful close contact is uncertain, since these distances are longer than the sum of the van der Waals radii (3.59 Å) of I and O atomic basins; *r_vdW_*(I) = 2.04 Å and *r_vdW_*(O) = 1.55 Å) [52]. The contact associated with the bond distance, 3.789 Å, is, however, relatively more linear than the remaining two, indicating that directionality alone does not necessarily correlate with interaction distances in this system.

As above, the F···I contacts are also more directional, whereas the F···O contacts are less directional and weaker; these may be regarded as secondary fluorine-centered close contacts. The F···I interaction with the periodate anion is aligned along the I–O bond direction of IO_4_^−^, and natural bond orbital analysis indicates charge transfer from the lone pair on fluorine to the σ*(I–O) antibonding orbital [13]. This implies that iodine, despite possessing a negative σ-hole, functions as the electron-density-accepting center, while fluorine behaves as the donor. However, the fluorine atom in the 3-fluoropyridinium cation is electrophilic and carries a positive electrostatic potential (*V_S,min_* = 56.4 and 67.7 kcal mol^−1^ in SMD and gas phase, respectively), thereby F···I is a fluorine-centered halogen bond.

While the F···I contact is negative σ-hole-driven and centered on the iodine, it does not correspond to a conventional halogen bond as the iodine atom acting as the interaction center is not electrophilic. The attraction nevertheless follows a coulombic picture involving an electrophilic region on fluorine interacting with a nucleophilic region associated with iodine, with orbital overlap further stabilizing the directional F···I contact. The close contact is thus best described as a directional, fluorine-centered halogen-bonding interaction, governed primarily by donor–acceptor overlap rather than by an electrophilic σ-hole on iodine. Similarly, the F···O interactions observed in the crystal are also regarded as fluorine-centered halogen-bonding interactions.

The interactions above are not the only intermolecular interactions responsible for the packing of molecules in the crystal but play a crucial role in the development of the I···O ordering between the anions leaning to the (IO_4_^−^)_n_ chain-like feature in the extended crystal. The C–H···O and N–H···O hydrogen bonds constitute the primary interactions, with the latter being apparently stronger than the former (*r* = 2.10–2.415 Å vs. 2.325–2.67 Å). Each cation interacts with several periodate anions, resulting in the (C_5_N)π···O contacts (*r* = 3.142 Å). These and the ones above together help to hold multiple anions in place.

**Figure 10:**
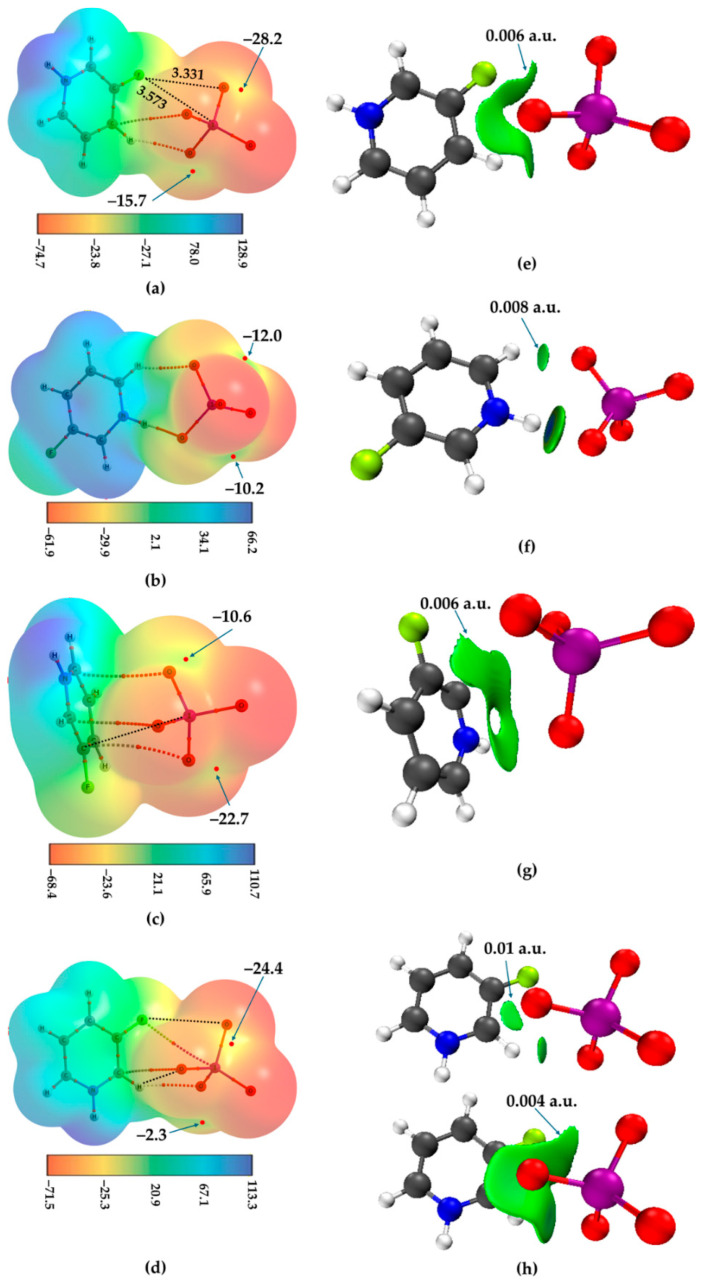
(**a**–**d**) QTAIM molecular graphs superimposed on MESP surfaces for four configurations of C_5_H_5_FN^+^···IO_4_^−^. Selected σ-holes (*V_S,max_*, in kcal mol^−1^) are indicated by small red circles and arrows. Black dotted lines in panels (**a**,**c**,**d**) are manually drawn to highlight interactions not captured by the QTAIM topology between certain atomic basins. (**e**–**h**) Corresponding IGMH isosurface plots for the dimers, with the isovalues specified in each panel representing the plotted surfaces.

Four models of the C_5_H_5_FN^+^···IO_4_^−^ dimer examined using the SMD approach show that iodine in IO_4_^−^ bears two or three σ-holes on its surface (Figure 10a–d). For instance, for Conf1 in Figure 10a, the *V_S,max_* values associated with the three σ-holes are −15.7, −28.2, and −31.9 kcal mol^−1^, while the global maximum of the potential is located on the protonated H atom (*V_S,max_* = 128.9 kcal mol^−1^), indicating that the anion acts entirely as a Lewis base. For Conf2 in Figure 10b, two σ-holes are observed, both remaining negative, with *V_S,max_* values of −10.2 and −12.2 kcal mol^−1^. The electrophilic character of the covalently bonded fluorine in the cation remains unchanged, with *V_S,min_* = 15.7 kcal mol^−1^.

The geometric stability of Conf1 arises from a combination of C···O, H···O, F···I, and F···O close contacts, with intermolecular distances (angles) of 2.442 Å (C–H···O = 151.0°), 3.218 Å (C=C···O = 118.5°), 3.573 Å (O–I···F = 172.7°; C–F···I = 103.5°), and 3.331 Å (C–F···O = 131.7°), respectively. The first interaction is σ-hole centered, the second is π-density (C) centered, the third involves a negative σ-hole, and the fourth is F-centered. QTAIM analysis captures the first two interactions but fails to reveal the latter two. Notably, the F···I close contact (marked by a dotted line in black) represents a fluorine-centered unconventional halogen bond, even though the F···I–O angle is quasi-linear and is confirmed by the IGMH plot shown in Figure 10e.

Second-order analysis suggests that the C–H···O interaction in Conf1 is described by LP(3) O → σ*(C–H) and LP(1) O → σ*(C–H) delocalizations, with *E*^(2)^ values of 1.70 and 0.34 kcal mol^−1^, respectively. The C···O interaction is characterized by the LP(3) O → BD*(2) (C=C) donation, with an *E*^(2)^ value of 0.84 kcal mol^−1^. The F···O contact is associated with LP(3) O → σ*(C–F) interaction, with a relatively small *E*^(2)^ value of 0.05 kcal mol^−1^, indicating its weak nature. No meaningful hyperconjugative contribution is associated with the F···I contact, as only a negligible BD*(1) (I–O) → RY*(4) (F) interaction is identified (*E*^(2)^ = 0.06 kcal mol^−1^).

For Conf2 (Figure 10b), the N–H···O and C–H···O interactions stabilize the geometry, with bond distances and angles of 1.778 Å (N–H···O = 178.6°) and 2.395 Å (C–H···O = 143.4°), respectively. These values indicate that the hydrogen bond formed by the protonated N–H of the organic cation is stronger than the C–H···O interaction, evidence of the bond paths that are described by a solid and a dotted line, respectively. These interactions are described by LP(3) O → σ*(N–H) and LP(1) O → σ*(C–H)/LP(3) O → σ*(C–H) with the *E*^(2)^ values of 19.17 and 0.54/1.10 kcal mol^−1^, respectively, and are in line with the IGMH isosurface plot shown in Figure 10f.

For Conf3, shown in Figure 10c, three negative σ-holes were observed on iodine, with potentials of −10.6, −15.2, and −22.7 kcal mol^−1^, while the fourth σ-hole is quenched upon engagement with the cation. QTAIM does not reveal any bonding topology between iodine and the C=C framework or the π-system of the arene ring; however, geometric analysis and IGMH results indicate a non-negligible C···I interaction (C···I = 3.726 Å, ∠C=C···I = 92.3°, and ∠C···I–O = 174.1°), as evidenced by the continuous isosurface connecting iodine and the C=C framework (Figure 10g). QTAIM does show three bond paths between the C framework of the C_5_N ring and the three oxygen atoms of IO_4_^−^, corresponding to three C···O tetrel bonds (*r* = 3.280, 3.343, and 3.406 Å). The C···I interaction is described by BD*(2) (C=C) → σ*(I–O), with an associated *E*^(2)^ value of 0.07 kcal mol^−1^. In addition, several LP(3) O → σ*(C=O) delocalizations, with *E*^(2)^ values in the range of 0.2–0.4 kcal mol^−1^, are identified, indicating the presence of a π-hole interaction.

Conf4, shown in Figure 10d, represents another minimum-energy configuration in which QTAIM analysis reveals the presence of F···I, C···O, and H···O close contacts, all of which are primarily coulombic in nature. The F···I contact corresponds to a fluorine-centered halogen bond, while the C···O and H···O interactions can be described as a carbon-centered tetrel bond and a hydrogen bond, respectively. The associated geometric parameters are as follows: *r*(F···I) = 3.615 Å, ∠C–F···I = 99.6°, and ∠F···I–O = 179.7°; *r*(C···O) = 3.110 Å with ∠N=C···O = 110.3°; and *r*(H···O) = 2.414 Å with ∠C–H···O = 142.8°. In addition to these interactions, intermolecular separations of 2.959 Å and 3.438 Å suggest the possible presence of additional H···O and F···O close contacts between the monomers. The charge-transfer delocalizations LP(3) O → σ*(C–H) and LP(3) O → σ*(C=C), with corresponding *E*^(2)^ values of 1.89 and 0.12 kcal mol^−1^, describe the primary C···O and H···O interactions, respectively. An additional hydrogen bond is associated with LP(3) O → σ*(C–H), with an *E*^(2)^ value of 0.44 kcal mol^−1^. No significant hyperconjugative interactions were detected to account for the F···O and O···I contacts.

The IO_4_^−^ anion in Conf4 exhibits three σ-holes on iodine along the O–I bond extensions, all of which are negative (*V_S,max_* = −2.3, −24.4, and −27.7 kcal mol^−1^). This indicates that complexation significantly polarizes the anion, reducing the magnitude of the negative electrostatic potential relative to the isolated monomer. Within the geometric framework of Conf4, the covalently bound iodine cannot be regarded as a halogen-bond donor; instead, it retains its nucleophilic character and is more likely to act as a base in the presence of an additional cationic unit.

The IGMH isosurface for Conf1 is irregular yet continuous (Figure 10e), encompassing all intermolecular interactions at an isovalue of 0.006 a.u. Increasing the isovalue to 0.01 a.u. retains only the C···O tetrel bond and the H···O hydrogen bonds identified by QTAIM, indicating that weaker interactions, such as F···O and C–H···O contacts, are observable only at lower isovalues and are not captured by QTAIM. A similar trend is observed for Conf2, where weak interactions are visible at an isovalue of 0.008 a.u. but vanish upon increasing the isovalue to 0.011 a.u. (Figure 10f). For Conf3, all C···O and C···I tetrel interactions are evident at an isovalue of approximately 0.006 a.u. (Figure 10g). The dominant interactions in Conf4 are the H···O and C···O contacts, as their associated isosurfaces persist at higher isovalues (∼0.01 a.u.) (Figure 10h, top). In contrast, O···I, F···O, and the remaining hydrogen-bond interactions are secondary in nature, appearing only at lower isovalues (<0.006 a.u.), as reflected by the emergence of their corresponding isosurface volumes (Figure 10h, bottom).

### 4.5. Case V

**Figure 11:**
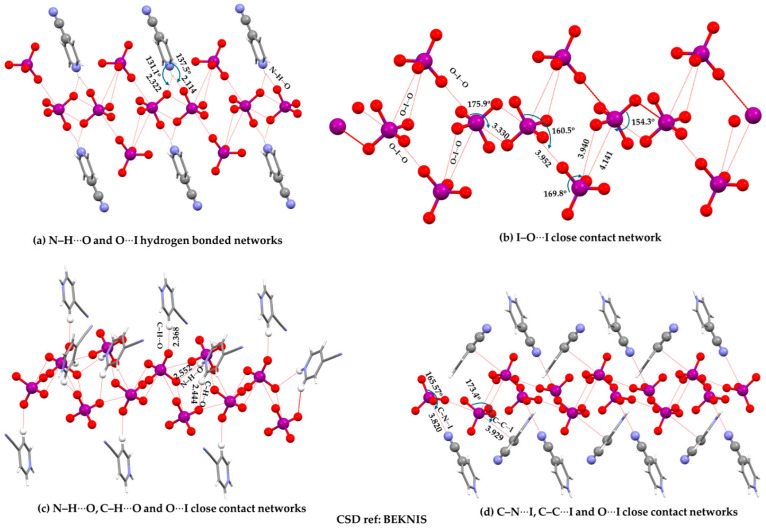
(**a**–**d**) Selected close contacts between interacting monomers that contribute to the development of O···I close contacts and the local ordering of IO_4_^−^ anions in the crystal structure of [C_6_H_5_N_2_^+^·IO_4_^−^]. Panels highlight: (**a**) N–H···O contacts; (**b**) O···I close contacts; (**c**) C–H···O and N–H···O contacts; (**d**) C···I and (**d**) N···I close contacts. Other close contacts are feasible. Bond lengths are given in Å, and bond angles in degrees.

The above rationalization is also applicable to the crystal structure of 4-cyanopyridinium periodate [C_6_H_5_N_2_^+^·IO_4_^−^] (CSD ref: BEKNIS) shown in Figure 11a–d. In this structure, specific hydrogen bonds such as N–H···O and C–H···O, together with π···O and N···I close contacts, cooperatively interplay to hold the anions in place, and these constitute the primary stabilizing contacts. The discussion presented in the original study [13] emphasizes mainly the anion–anion interactions, which are relatively faint and contribute only marginally to the development and stability of the crystal.

The observed intermolecular ordering between the anions is therefore not just governed by O···I close contacts. These contacts occur in three sets: one equivalent pair that is relatively shorter (*r* = 3.330 Å), and another set that is inequivalent and longer (*r* = 3.940 and 4.141 Å), together with an additional contact at 3.952 Å (Figure 11b). Collectively, these long-range contacts form a pseudo-rhomboidal local network comprising four IO_4_^−^ units in 2D.

Each (IO_4_^−^)_2_ pair is surrounded by several organic cations. Figure 11c illustrates only the N–H···O (*r* < 2.35 Å) and C–H···O (2.35 < *r* < 2.60 Å) hydrogen-bonding networks and the manner in which they hold the anions together. In these interactions, the anion acts primarily as a nucleophilic partner, and the N–H···O contacts are relatively stronger than the C–H···O ones. It is also worth noting that another type of N–H···O contact is present that is somewhat longer, with *r* = 2.552 Å. Increasing the H···O distance up to the sum of the van der Waals radii of H and O (2.75 Å) reveals additional close contacts between the anion and cation; however, these are expected to be weaker and may be regarded as van der Waals-type interactions.

Two other dominant chemical synthons in the crystal are the C···I and N···I close contacts, in which the former are longer than the latter, consistent with the van der Waals radii of C (1.77 Å [52]) and N (1.66 Å [52]). These contacts can be classified as π-centered tetrel bonds and electron-deficient p-type pnictogen bonds, respectively. Clearly, whether the O···I close contacts between the anions represent true halogen bonds remains unclear. They can, however, be regarded as anion–anion interactions, although it is not straightforward to classify them as either anti-electrostatic or electrostatically driven, given that it is not clear whether the iodine in the anion possesses an electrophilic region. This assessment is also based on the observation that the ordering of the anions is largely dictated by the multiple hydrogen-bond contacts and other interactions, which are predominantly coulombic in nature.

**Figure 12:**
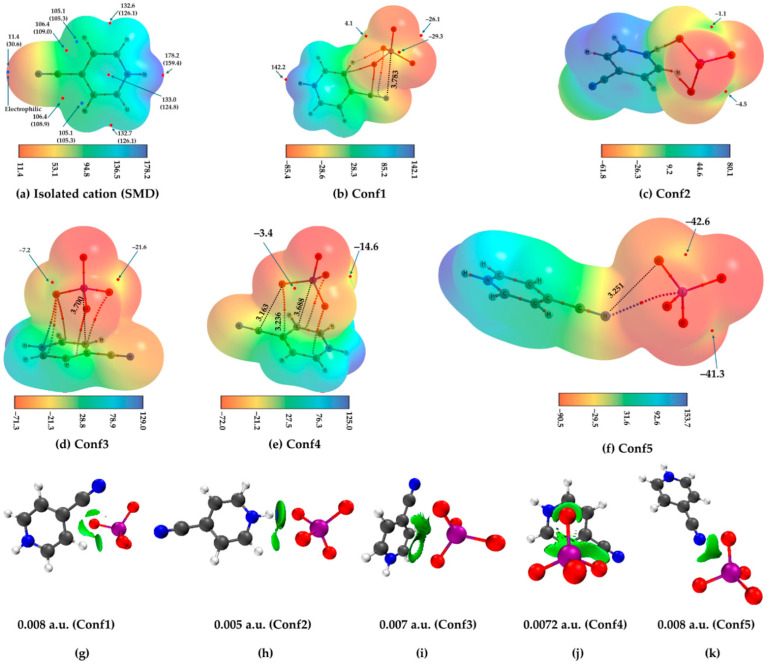
(**a**) SMD-level QTAIM topology superimposed on the MESP plot of the organic cation in the crystal structure of [C_6_H_5_N_2_^+^·IO_4_^−^]; values (kcal mol^−1^) in parentheses correspond to gas-phase results. The potential was mapped on the 0.001 a.u. electron density isosurface. (**b**–**f**) Corresponding plots for the C_6_H_5_N_2_^+^···IO_4_^−^ dimers, where the potential was mapped on the 0.0013 a.u. isodensity envelope. Selected close-contact distances (values in Å) not identified by bond path and bond critical point topologies are indicated by black dotted lines. (**g**–**k**) IGMH isosurface plots of the corresponding complexes, with the isovalues indicated in each panel.

The views developed above are consistent with the monomer and dimer model calculations. As shown in Figure 12a, the nitrogen atom in the cation is entirely electrophilic, regardless of whether a solvated or gas-phase geometry is used, and no σ-hole is present along the extension of the –C≡N cyanogen group; the electron depletion on its surface arises primarily from the cationic nature of the molecule. This is reflected in the potential at N, where a positive minimum is observed. The strength of the minimum is relatively weaker in solution than in the gas phase (*V_S,min_* = 11.4 vs. 30.6 kcal mol^−1^). We also observed a shift in the location of the minimum: in the gas phase, it appears nearby, on the surface of C of the arene framework, above and below the plane, whereas in solution it moves to a slightly different region in close vicinity. This shift arises from solvent polarization effects, which redistribute the electrostatic potential and partially screen the cationic charge, illustrating how environmental factors influence local electrophilicity.

In Conf1, Figure 12b, two σ-holes on iodine identified on the 0.001 a.u. isodensity envelope are negative, with *V_S,max_* values of −30.5 and −30.6 kcal mol^−1^. The third σ-hole, missing along the extension of the remaining O–I bond in IO_4_^−^ at this envelope, becomes visible when a 0.0013 a.u. isodensity surface is used. On this surface, the three σ-holes have strengths of 4.0, −26.1, and −29.2 kcal mol^−1^, respectively. The stability of this complex arises from H···O and (C≡N)_π_···I interactions. There are no other significant coulombic contacts. In this case, the negative σ-hole on iodine in IO_4_^−^ acts as a nucleophilic site toward the electron-deficient π-density of the cyano group. The corresponding O–I···N and O–I···C angles are 178.3° and 163.8°, respectively, indicating a high degree of directionality.

Five different configurations extracted from the crystal were energy-minimized in the solution phase. Figure 12b–f shows the QTAIM-superimposed MESP plots of the five ion pairs that mimic the nature of packing of monomers in the crystal.

The (C≡N)_π_···I interaction is better described as a π-centered pnictogen-bonding interaction, rather than a halogen bond. The second-order NBO analysis yields small stabilization energies of *E*^(2)^ = 0.05–0.17 and 0.44/1.89 kcal mol^−1^ for the LP(1)/(2)/(3) (O) → BD*(2)/(3) (C≡N) and LP(1)/(3) O → σ*(C–H) delocalizations, respectively, confirming the pnictogen and hydrogen bonds, respectively. However, no charge–transfer interaction such as LP(N) → σ*(I–O), or any related donor–acceptor delocalization, was detected. The N···I attraction inferred from the geometry, which is supported by the IGMH (Figure 12g) and MESP analyses, is not reflected in the NBO description, and QTAIM failed to identify the same for the complex. Whether this discrepancy arises from the limitations of the NBO approach in capturing such weak noncovalent interactions remains unclear.

A similar behavior is observed for Conf2, as shown in Figure 12c, in which the two σ-holes on the iodine atom of IO_4_^−^, located opposite to the cation direction, are negative (*V_S,max_* = −6.0 (−1.4) and −9.5 (−4.5) kcal mol^−1^ when mapped on the 0.001 (0.0013) a.u. electron density isosurface). The maxima on the protonated H atoms are annihilated upon engagement with an O site of the same cation. QTAIM analysis indicates the presence of this interaction with *r*(H···O) = 1.757 Å and ∠N–H···O = 172.5°, as well as another longer-range contact with *r*(H···O) = 2.872 Å and ∠C–H···O = 114.6°. The second-order NBO analysis suggests that the strengths of the former and latter hydrogen bonds can be quantified by *E*^(2)^ values of 5.73 and 0.14 kcal mol^−1^, associated with the charge-transfer delocalizations LP(1) O → σ*(N–H) and LP(3) O → σ*(C–H), respectively, indicating that the latter interaction is predominantly of van der Waals type. It is worth noting that a weak back-donation from BD(1) N–H to RY*(7) O and σ*(I–O) is also observed for the former interaction with *E*^(2)^ values of 0.11 and 0.07 kcal mol^−1^, respectively.

For Conf3, as shown in Figure 12d, the optimized geometry exhibits a C_π_···I close contact with *r*(C···I) = 3.700 Å and ∠C···I–O = 178.0°, indicative of a tetrel interaction. This is described by σ(I–O) → RY*(C) donation, with an *E*^(2)^ value of 0.08 kcal mol^−1^. An (H)N···O contact is also present within the dimeric framework, with *r*(N···O) = 3.306 Å and ∠H–N···O = 107.2°, which may be consistent with a pnictogen-type interaction. However, this contact is characterized by LP(2) O → BD*(2) (N=C) and LP(3) O → BD*(2) (N=C) delocalizations, with corresponding *E*^(2)^ values of 0.07 kcal mol^−1^, indicating a very weak hyperconjugative contribution. Two additional C–H···O close contacts may be revealed with H···O distances of 3.283 and 3.293 Å, suggestive of weak hydrogen bonds.

QTAIM confirms only the pnictogen bond and does not recover bond paths for the tetrel or the two hydrogen bonds. Instead, it assigns bond paths corresponding to (C–C)_midpoint_···O tetrel-type interactions and reveals two additional bond paths involving the carbon atoms bonded to the nitrogen of the protonated amine, with a concomitant charge transfer with an *E*^(2)^ of 0.40 kcal mol^−1^. Clearly, the latter interaction type may suggest the presence of a (C_5_N)(π-hole)···O-type engagement. Although this configuration closely resembles that observed in the crystal, the MESP results show that one σ-hole is annihilated upon interaction with the cation, while the remaining three remain negative regardless of whether the 0.001 or 0.0013 a.u. electron density isosurface is used to map the potential. The corresponding $V_{S,max}$ values are −11.0 (−6.9), −11.5 (−7.2), and −26.2 (−21.6) kcal mol^−1^, respectively, where the parenthesis values correspond to the 0.0013 a.u. envelope.

Conf4, shown in Figure 12e, is geometrically very similar to Conf3, but in the former the iodine atom of IO_4_^−^ is oriented toward a carbon atom of the arene ring, forming a directional C···(σ-hole)I tetrel-bonded close contact (*r* = 3.688 Å and ∠C···I–O = 174.8°, ∠H–C···I = 93.3° and ∠C=C···I = 87.4°). QTAIM does not confirm this interaction and instead reveals three bond paths between the molecular entities: one C···O and a pair of (C=C)···O close contacts. None of these can be associated with a π-hole, as no $V_{S,\max}$ is observed on the arene carbon or on the delocalized C=C bond of the C_6_N arene moiety, yet these contacts are predominantly coulombic.

From a geometric standpoint, the C···O assignment is not well defined, since the O atom is directed toward the (C=C)_midpoint_ of the fragment associated with the cyanogen group; the corresponding C···O distances involving the arene carbon and the cyanogen carbon are 3.236 and 3.163 Å, respectively. These are tetrel bonds. The iodine atom exhibits three σ-holes along the O–I bond extensions with $V_{S,\max}$ values of −7.8 (−3.4), −19.2 (−14.6), and −13.2 (−8.7) kcal mol^−1^, of which the first two are shown in Figure 12e, where the parenthesis values represent the 0.0013 a.u. electron density isosurface. The remaining one is annihilated during the formation of the C···I close contact.

The NBO analysis reveals several very weak interactions between the monomers. Among these, LP(2)/LO(2) O → π*(C≡N) with *E*^(2)^ = 0.29/0.16 kcal mol^−1^ is observed, which could be interpreted as a weak orbital contribution but does not correspond to a well-defined tetrel or pnictogen bond. Additionally, an apparent interaction between the ring C=N and an oxygen lone pair (*E*^(2)^ = 0.53 kcal mol^−1^) is detected; however, this oxygen is not properly oriented toward the bond, indicating that this delocalization is not physically meaningful. No significant charge transfer is observed between the C and I atoms that would indicate the C···I tetrel bond. A minor hyperconjugation from BD*(1) I–O to RY*(4) C (*E*^(2)^ = 0.16 kcal mol^−1^) is detected, but its contribution is very small and cannot be considered as stabilizing a classical tetrel interaction.

QTAIM predicts a single bond path between I and N in Conf5, Figure 12f, indicating an N···(σ-hole)I close contact (*r* = 3.441 Å, ∠C≡N···I = 146.0°, and ∠N···I–O = 178.0°). This corresponds to a pnictogen-type contact involving the electrophilic nitrogen and the negative σ-hole on iodine, rather than a conventional halogen bond. The deviation from linearity in ∠C≡N···I is likely due to a secondary (C≡)N···O interaction, with *r*(N···O) = 3.251 Å and ∠C≡N···O = 116.0°. The second-order NBO analysis indicates hyperconjugative interactions BD(1) (C≡N) → RY*(10) I and LP(1) N → σ*(I–O) with *E*^(2)^ values of 0.05 and 0.1 kcal mol^−1^, respectively, confirming the unconventional N···(σ-hole)I pnictogen bond. Additional hyperconjugations are observed between the second and third lone-pair orbitals of the three oxygen atoms facing the cyanogen group of the cation and the antibonding σ* and π* orbitals of C≡N, with *E*^(2)^ values ranging from 0.05 to 0.40 kcal mol^−1^. Among these, the N···O contact (*r* = 3.251 Å) exhibits the strongest *E*^(2)^ of 0.40 kcal mol^−1^.

The MESP plot further corroborates the presence of the N···(σ-hole)I and N···O interactions. The transformation of the negative σ-holes on iodine into positive $V_{S,\max}$ regions is not observed upon the interaction of IO_4_^−^ with the cation. The three remaining σ-holes on iodine retain negative $V_{S,\max}$ values of −45.3 (−41.3), −46.5 (−42.6), and −51.7 (−47.8) kcal mol^−1^, where parentheses values correspond to those evaluated on the 0.0013 a.u. isodensity envelope.

The geometrically and MESP-assigned close contacts identified for Conf1–Conf5 are consistent with the IGMH isosurface plots shown in Figure 12g–k, including those interactions that were not captured by the QTAIM and NBO analyses. The trend in *E_b_* follows this order across the dimer series: Conf2 (−5.91 kcal mol^−1^) > Con3 (−5.27 kcal mol^−1^) > Conf4 (−5.13 kcal mol^−1^) > Conf1 (−3.77 kcal mol^−1^) > Conf5 (−1.61 kcal mol^−1^).

## 5. Crystals in Different Dimensions

The zero-dimensional (0D) nature of the IO_4_^−^ anion has been observed in several crystals. Representative examples include: [C_10_H_12_S_4_^+^·C_10_H_12_S_4_·IO_4_^−^] (CSD ref: DOFNAN01), [C_30_H_32_IrN_4_O_4_^+^·IO_4_^−^·CCl_4_] (CSD ref: KEJTAX), [C_12_H_24_O_6_·C_6_H_7_N_2_O_2_^+^·IO_4_^−^] (CSD ref: NEGVAZ01), [C_18_H_26_N_2_P^+^·IO_4_^−^] (CSD ref: ROSDOV), [C_15_H_29_N_3_P^+^·IO_4_^−^] (CSD ref: ROSFAJ), tetrakis(isopropylamino)phosphonium periodate [C_12_H_32_N_4_P^+^·IO_4_^−^] (CSD ref: ROSFIR), and tetraphenylarsonium periodate [C_24_H_20_As^+^·IO_4_^−^] (CSD ref: SOVYIL). In all of these structures, H···O hydrogen bonds with distances below 2.5 Å act as the primary synthons stabilizing the crystal lattice.

**Figure 13:**
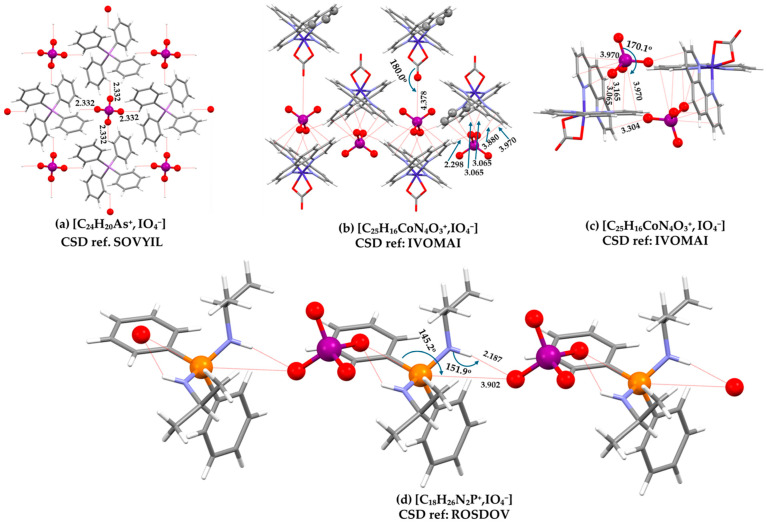
(**a**–**d**) Illustration of the 0D nature of the IO_4_^−^ anion in selected crystal structures deposited in the CSD. Selected bond distances and bond angles are given in Å and degrees, respectively. The organic cation in (**c**) is deleted for clarity.

Figure 13a,b show three representative crystals in which the IO_4_^−^ anions appear as 0D. In Figure 13a, H···O hydrogen bonds serve as the primary connectors between the monomers in the crystal structure of [C_24_ H_20_As^+^,IO_4_^−^], whereas in Figure 13b, which displays the crystal structure of [C_25_H_16_CoN_4_O_3_^+^,IO_4_^−^], both (C=C)π···O and H···O interactions are the dominant stabilizing contacts. In the latter case, the attraction between the carbon framework of the arene moiety and the oxygen atom of the anion cannot be neglected, as the intermolecular distances (*r* < 3.2 Å) are shorter than the sum of the van der Waals radii of carbon and oxygen, suggesting the presence of π···O interactions. The (C=C)π···I contacts measure 3.970 and 3.880 Å, with corresponding I–O···C angles of 170.1° and 159.4°, respectively. These are the result of two negative σ-holes on the iodine atom of the anion engaged as bases to the two phenanthroline moieties, giving rise to directional interactions that are tetrel bonds. All of these interactions are primarily coulombic in nature, as the positive sites on the cation interact with the negative σ-holes on the anion, and should not be recognized as halogen bonds. Moreover, a directional C–O···I proximity is also observed; however, its significance as a meaningful close contact is questionable because the O···I distance (*r* = 4.378 Å and ∠C–O···I = 180.0°) is considerably longer than the sum of the van der Waals radii of iodine and oxygen (3.59 Å). Therefore, it cannot reliably be recognized as a chalcogen bond.

The crystal of [C_25_H_16_CoN_4_O_3_^+^, IO_4_^−^], shown in Figure 13c, provides a representative example in which tetrel bonding, together with H···O hydrogen bonding, governs the packing of the molecular entities. The tetrel interactions are primarily of the π-hole···O and (C=C)_π_···O types, occurring within the 3.0–3.4 Å distance range, whereas the C–H···O hydrogen bonds fall within 2.2–2.6 Å. In addition, I···O close contacts between the anion and cation should not be overlooked, as they are directional (*r*(I···O) < 4.0 Å), with ∠O–I···O angles approaching 170.1°, indicating a structured, though weak, interaction.

Figure 13d represents another such crystal structure, [C_18_H_26_N_2_P^+^·IO_4_^−^], in which the IO_4_^−^ anion is only 0D but also acts as an electron-density donor to electrophilic sites. In addition to the network of H···O hydrogen bonds, the N–H···O interactions constitute the primary chemical synthons, while the P···O close contacts formed between the cation and anion are comparatively less directional. The P···O separation is 3.902 Å, which is appreciably longer than the sum of the van der Waals radii of P (1.90 Å) and O (1.55 Å, total 3.45 Å), indicating that this interaction is weak and predominantly electrostatic in nature.

**Figure 14:**
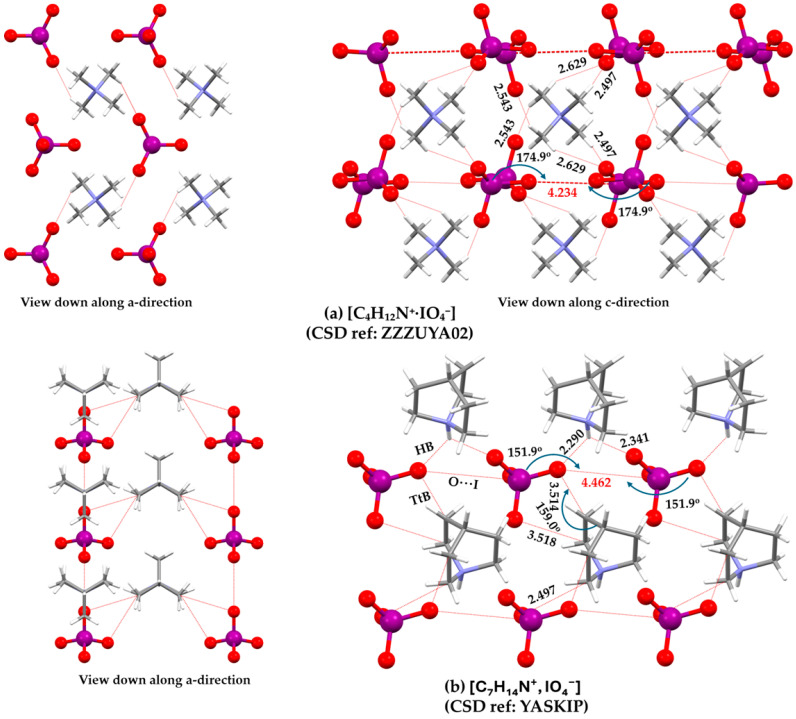
(**a**,**b**) Illustration of directional O···I close contacts in selected crystals that give the IO_4_^−^ anions a one-dimensional (1D) arrangement, in which the intermolecular distances are generally longer than the sum of the van der Waals radii of O and I. In these crystals, hydrogen bonding and other interactions play a critical role in the packing of the molecular entities. Selected bond distances and bond angles are given in Å and degrees, respectively.

Several other crystal structures have been reported in which the IO_4_^−^ anions appear to be 0D. However, when intermolecular distances between adjacent periodate anions that are slightly longer than the sum of the van der Waals radii of iodine and oxygen are considered, a one-dimensional (1D) ···IO_4_^−^···IO_4_^−^···IO_4_^−^ chain-like feature emerges, in which the IO_4_^−^ units are arranged in a back-to-back manner. This gives rise to a pseudo-1D motif, as illustrated in Figure 14a,b for the crystals of tetramethylammonium metaperiodate [C_4_H_12_N^+^·IO_4_^−^] (CSD ref: ZZZUYA02) and 1-azoniabicyclo[2.2.2]octane periodate [C_7_H_14_N^+^, IO_4_^−^] (CSD ref: YASKIP).

An extensive network of ordered H···O hydrogen bonds holds the anionic moieties together in a compact configuration in [C_4_H_12_N^+^·IO_4_^−^], as shown in Figure 14a. These contacts are weak, with H···O distances in the range 2.50–2.70 Å, yet they fall within the sum of the van der Waals radii of H and O (2.75 Å; H = 1.20 Å, O = 1.55 Å) and therefore cannot be disregarded. By contrast, the I···O contacts are long-ranged, with *r*(I···O) = 4.234 Å and nearly linear O–I···O and I···O–H angles of 174.9°. These features indicate that the I···O interactions are extremely weak and secondary, arising as a forced geometric consequence of the hydrogen-bonding network rather than acting as primary structure-directing interactions. Indeed, this distance is appreciably larger—by approximately 26%—than the I···O halogen-bond distance (*r* = 3.363 Å, C–I···O = 159.1°) observed between monomeric units in the crystal structure of 1,1,1-triacetoxy-1λ^5^,2-benziodoxol-3(1H)-one (CSD refcode: ZAZJOB), which does not have any anion.

A similar argument may be advanced for [C_7_H_14_N^+^·IO_4_^−^], shown in Figure 14b. In this case, in addition to the extensive network of O···H hydrogen bonds (*r* ≤ 2.4 Å) between the interacting entities, C···O tetrel-bonding interactions also act as structure-directing motifs that are more directional than the O···I contacts and most of the hydrogen bonds. The O···I separations (*r* = 4.462 Å) are significantly longer than those found in the ion pair discussed above, indicating that these contacts are even weaker and likely arise as secondary, geometry-imposed features of the hydrogen- and tetrel-bonding network.

**Figure 15:**
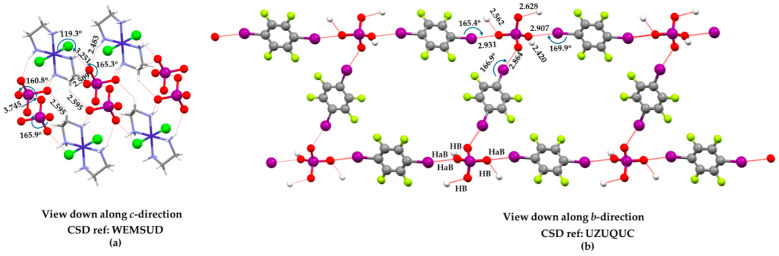
(**a**,**b**) Zero-dimensional (0D) nature of the IO_4_^−^ anion in two selected crystals, illustrating selected intermolecular close contacts (bond lengths in Å and bond angles in degrees). Intermolecular contacts, including the hanging ones, are shown as dotted lines. HB and HaB in (**b**) refer to hydrogen- and halogen-bonds, respectively.

The intermolecular geometric parameters in [C_4_H_16_Cl_2_CoN_4_^+^·IO_4_^−^], as shown in Figure 15a, satisfy both directionality and distance criteria, with O···Cl = 3.251 Å and ∠I–O···Cl = 165.3°, and the separation is shorter than the sum of the van der Waals radii of Cl and O (3.37 Å; Cl = 1.82 Å, O = 1.55 Å). What can be said about the O···Cl close contact in the crystal structure of [C_4_H_16_Cl_2_CoN_4_^+^·IO_4_^−^]? It is unclear whether this contact should be described as a halogen bond or a chalcogen bond. To answer this, we keep in mind that the transition-metal complex is cationic, and the Co–Cl-bound chlorine atom is electrophilic, with positive electrostatic potential distributed along both the axial extension of the Co–Cl bond and the lateral regions. As a result, chlorine acts as a halogen-bond donor, while the oxygen atom of the periodate anion functions as the acceptor. The resulting O···Cl contact is therefore better described as a Cl···O halogen bond rather than a chalcogen-bonding interaction. Moreover, a minimally extended hydrogen-bonding network (with H···O distances below ~2.6 Å) between the IO_4_^−^ anions holds them in a compact arrangement, locally generating (IO_4_^−^)_2_-like motifs. These are directional, and the O···I separations are shorter than twice the van der Waals radius of iodine, further supporting the idea that the anion–anion arrangement is enforced by hydrogen bonding, while the O···Cl halogen bond contributes as an additional structure-stabilizing interaction.

The crystal structure of [1.5(C_6_F_4_I_2_)·IO_4_^−^·C_16_H_36_N^+^] (CSD ref: UZUQUC), shown in Figure 15b, exhibits a distinctive set of intermolecular interactions. One set of I···I and I···O close contacts is observed, with separations of 3.999 and 2.907 Å, respectively, and corresponding C–I···I and C–I···O angles of 156.3° and 169.9°, respectively. Both contacts are directional, although the latter is significantly more linear than the former. This suggests that the primary interaction is the C–I···O close contact rather than the C–I···I contact, arguably because the lateral portion of O along I–O bond extensions in IO_4_^−^ is relatively more electrophilic than the σ-hole on I along O–I bond extensions. It should be noted that not all nucleophilic or electrophilic regions in a molecule necessarily engage in intermolecular interactions of opposite polarity when brought into proximity; instead, the arrangement that maximizes attractive engagement is favored [53]. In this case, the I···O interaction is stronger, and the geometry adjusts accordingly, causing the I···I contact to deviate from linearity. Whether the latter should be regarded as a meaningful intermolecular interaction requires further detailed analysis. Nevertheless, hydrogen bonds between the organic cation and Lewis-basic oxygen atoms also play an important role in stabilizing the overall crystal structure.

It should also be emphasized that the iodine atom of IO_4_^−^ cannot be considered a halogen-bond donor (acid) in this system. Three oxygen atoms of the periodate anion are engaged via the C–I···O links with three C_6_F_4_I_2_ molecules, forming three inequivalent interactions that are halogen bonds, while these and the remaining oxygen atom participates in an H···O hydrogen bond with a methyl group of the organic cation. Thus, IO_4_^−^ acts exclusively as an acceptor of electrophilic interactions rather than as a donor, and is 0D.

## 6. Discussion and Conclusions

Several crystal structures are examined as representative examples, and selected intermolecular interactions are highlighted to demonstrate their importance in ionic assemblies, as well as the occurrence of halogen bonding in systems where the halogen center is ubiquitously electrophilic. In other cases, similar directional interaction patterns are observed along the extensions of O–I bonds; however, these are not regarded as halogen bonds.

The primary intermolecular interactions in crystals occur between cations and anions, whereas close contacts between anions or between cations generally represent secondary or tertiary interactions. Although these weaker interactions are not the principal drivers of crystal formation, they influence packing arrangements and therefore remain important for understanding and designing crystalline materials with tailored or enhanced functionalities.

An IO_4_^−^ anion is repulsive toward another such anion in the gas phase and therefore does not self-assemble. When combined with organic cations, the anion does not invariably undergo a transformation in which the highly negative σ-holes on iodine become positive through polarization induced by primary noncovalent interactions with the cation. While some dimer models display such transformations depending on the nature of the organic cation and the site of interaction, these models fail to capture the full complexity of the crystal environment. Consequently, they cannot be reliably used to demonstrate the halogen-bond-donating capabilities of the anions, as has been suggested in some studies [13].

Although these conclusions are based on computational results for selected ion-pair systems, it is important to emphasize that crystals rarely exhibit isolated anion–anion interactions in the absence of counterions. In solution, assemblies such as (IO_4_^−^)_n_ are stabilized by the solvent. The quasi-linearity of the O···I interactions in (IO_4_^−^)_n_ in such environments, together with the negative σ-hole-centered interactions associated with iodine, should not be recognized as halogen bonds [5]. Instead, these interactions are more appropriately described as anti-electrostatic interactions, enabled by the polar (high-dielectric) medium, which screens coulombic repulsion between the anions rather than reflecting true σ-hole-driven attraction.

O···O interactions, which sometimes appear secondary, cannot be straightforwardly interpreted as chalcogen bonds according to the conventional definition [7], which requires interaction between an electrophilic region on one covalently bonded chalcogen atom and a nucleophilic site on another. In the present systems, both interacting oxygen atoms are electron-rich. Although the observed O···O contacts often resemble a Type-II topology, they are far from linear; nonetheless, they may satisfy the geometric criteria typically associated with chalcogen bonding. Such contacts are feasible in many crystals but do not constitute chalcogen bonds, as they frequently arise from packing effects imposed by surrounding organic cations that bring the anions into close proximity. Whether these O···O close contacts are intrinsically stabilizing or destabilizing remains an open question.

NBO analysis can, in some cases, predict hyperconjugative interactions between hypothetical donor–acceptor fragments that are spatially distant in optimized dimer geometries. In several instances, it also fails to capture charge-transfer (hyperconjugative) interactions between interacting fragments, particularly in the description of O···I contacts.

We have explicitly shown, using SAPT, that the interaction energy of cation–cation dimers is strongly repulsive in the gas phase. The repulsive electrostatic component is consistent with what might be referred to as anti-electrostatic behavior, whereas the induction and dispersion contributions remain attractive. The same behavior is observed for anion–anion dimers. Assigning such interactions as σ-hole-centered hydrogen- or halogen-bonded interactions would sharply violate the definitions of hydrogen bonding [4] and halogen bonding [5,6]. In that case, the definitions put forward by the IUPAC working group for halogen, chalcogen, hydrogen, and pnictogen bonding, including those proposed by us for halogen, pnictogen, and tetrel bonds, may require reassessment in the context of such systems, as the observed arrangements do not exhibit the conventional electrophile–nucleophile complementarity that underpins classification of these interactions.

The observation that the σ-holes on iodine in IO_4_^−^ are entirely negative, which is less so compared to the covalently bound oxygen, and that the anion therefore cannot function as a conventional halogen-bond donor, is consistent with earlier discussions of fully fluorinated dimers. In such systems, negative σ-holes on fluorine along the extensions of C–F bonds participate in attractive yet directional F···F interactions to form (C_6_F_6_)_2_ dimers [35], in agreement with other studies showing that like-charged electrostatic sites can nevertheless engage in attractive interactions [51].

A recent study has suggested that halogen bonds may form even when the classical σ-hole–lone pair criterion is violated [20]. Although such contacts may be weakly attractive due to polarization and dispersion forces, they may not conform to the classical definition of a halogen bond, which requires an attractive interaction between an electrophilic region on the halogen in one species and a nucleophilic site on another. This broader interpretation connects to the ongoing discussion of interactions between like-charged species, which are frequently categorized as “anti-electrostatic” associations, although they may be more appropriately regarded as counterintuitive interactions [54,55,56,57]. It has been argued that the term “anti-electrostatic” may be misleading [58], since a complete Coulombic description of intermolecular interactions inherently includes polarization, dispersion, and environmental effects. In this view, the apparent association of like-charged regions of electrostatic potential is better described as counterintuitive, given that it involves interactions between positive···positive and negative···negative regions of electrostatic potential. Nevertheless, SAPT analysis typically reveals a repulsive electrostatic contribution for both anion–anion and cation–cation assemblies, with the overall stabilization arising from attractive induction and dispersion components.

There is no hard and fast rule that a σ-hole is needed on the halogen in the halogen-containing molecular entity in the isolated state, nor is a positive σ-hole a strict requirement, but the covalently bonded halogen atom has to be positive in order to donate a halogen bond. The possibility that a σ-hole is not always required for halogen bond formation was highlighted approximately a decade ago by Zhang and co-workers [59]. They described this a new class of halogen bonds of the type X = Hal···Y in complexes such as CH_3_OCZCl^+^···Y (Z = H, F, or Cl; Y = F^−^, Cl^−^, or Br^−^) as model systems. The strength of these interactions was reported to lie in the range 90–120 kcal mol^−1^, substantially exceeding that of conventional halogen bonds of the type X–Hal···Y. Natural bond orbital analysis indicated that LP → π* interactions govern both the directionality and stabilization of these contacts.

The present findings may also have implications for crystal engineering and the design of organic–inorganic materials containing halogen oxyanions. In particular, the results suggest that short and directional O···I contacts involving perhalate anions should not automatically be regarded as reliable structure-directing halogen-bonding motifs. Instead, the formation of the anion–anion assemblies appears to depend strongly on the surrounding ionic environment, dielectric screening, counterion effects, and collective crystal-packing forces. Consequently, the rational design of new materials incorporating halogen oxyanions may benefit from considering these environmental contributions explicitly, rather than relying solely on geometrical criteria or presumed σ-hole-driven donor–acceptor interactions.

## 7. Computational Details

The geometries of the monomers, dimers, and oligomers were extracted from crystal structures deposited in the Cambridge Structural Database (CSD) [28,60] and fully optimized using the M06-2X functional [61,62] with the def2-TZVPPD basis set, employing the Solvation Model based on Density (SMD) [41] with water as the solvent. All calculations were performed using the Gaussian 16 suite of programs [63], and the basis set was obtained from the EMSL Basis Set Library [64].

Gas-phase calculations were also performed for selected cases to assess the effect of solvation on geometries and binding energies. Frequency calculations were carried out for all systems, both in the gas phase and in solution, confirming that the geometries discussed belong to the stationary points.

The binding energies of the dimers were calculated as the difference between the total energy of the optimized complex and the sum of the total energies of the individually optimized monomers. For these calculations, the energy-minimized geometries of all species were used.

Quantum Theory of Atoms in Molecules (QTAIM) [42], Independent Gradient Model based on Hirshfeld partition (IGMH) [47], and Molecular Electrostatic Potential (MESP) [43,44,65] analyses were performed using wavefunctions obtained from the optimized geometries. While QTAIM was applied, we did not attempt to elaborate on the full bonding topology because in several cases it failed to identify all expected bond paths and bond critical points. Therefore, only the QTAIM molecular graphs are presented to illustrate which interactions are captured and which are not. The AIMAll [66], Multiwfn [67] and VMD [68] codes were used.

Different isovalues were employed in the IGMH analysis to visualize both weak and relatively strong intermolecular interactions. This approach also highlights whether the absence or presence of bond paths in QTAIM is misleading, as the strict zero-flux boundary condition used in QTAIM can fail to capture certain interactions. By varying the isovalues, IGMH provides a complementary view of the interaction regions that may not be fully revealed by QTAIM topology and NBO’s second-order energy [69] analyses.

The MESP approach was employed to examine the directional nature of positive and negative σ-holes on halogen atoms in anions and complexes, and to assess whether the resulting directional interactions between anions can be rationalized as conventional halogen bonds or should instead be referred to as “anti-electrostatic interactions,” without introducing the term “anti-electrostatic halogen bond.” The maxima and minima of the electrostatic potential on the molecular surfaces are referred to as $V_{S,\max}$ and $V_{S,\min}$, respectively. Unless otherwise stated, a 0.001 a.u. electron density isosurface was used to map the potential.

Symmetry-adapted perturbation theory (SAPT) [48,49] was applied to the SMD-optimized geometries, with computations performed in the gas phase since the formalism is not yet available for solution-phase calculations. SAPT decomposes the interaction energy into electrostatics, exchange repulsion, induction, and dispersion contributions. For small systems, the SAPT2+3(CCD) level was used, with dispersion evaluated at the CCD level, whereas larger dimers were treated at the SAPT0 level. The PSI4 code was used [70].

## Data Availability

This research used data reported in the manuscript itself.

## Supplementary Materials

The following supporting information can be downloaded at: https://www.mdpi.com/article/10.3390/molecules31122153/s1, Table S1: Geometrical parameters of the IO_4_–···O–X contacts identified from the Cambridge Structural Database (CSD) (version 6.01). The search employed an I···O distance criterion of 2.0–4.0 Å and an O–I···O angular criterion of 140–180°. Only single-crystal structures with R-factor ≤ 0.05 and no reported crystallographic errors were considered. The table lists the CSD refcodes together with the corresponding I···O distances, r, and O–I···O angles, ∠; Table S2: Statistical summary of the IO₄···O–X contacts identified from the Cambridge Structural Database (CSD). The search employed an I···O distance criterion of 2.0–4.0 Å and an O–I···O angular criterion of 140–180°. Only single-crystal structures with R-factor ≤ 0.05 and no reported crystallographic errors were included. The dataset comprises 49 close contacts extracted from 16 unique crystal structures.
