# Supplementary material for: Halogen Bonds or Not? Reassessing Noncovalent Interactions in Crystals of Periodate Anion from the Cambridge Structural Database
Source: Molecules. 2026 Jun 18;31(12):2153. doi: 10.3390/molecules31122153 (PMC13306011; doi:10.3390/molecules31122153)
Supplement: Supplementary file 1 [file molecules-31-02153-s001.zip › molecules-4364553-supplementary.pdf]

Halogen Bonds or Not? Reassessing Noncovalent Interactions in Crystals of Periodate Anion from the Cambridge Structural Database

Arpita Varadwaj, [1]*Pradeep R. Varadwaj, [1-3]*Helder M. Marques, [3]Ireneusz Grabowski, [2,4]Mohd. Mudassir Husain, [5]Koichi Yamashita [1]

1 Department of Chemical System Engineering, School of Engineering, the University of Tokyo 7-3-1, Tokyo 113-8656, Japan
2 Institute of Physics, Faculty of Physics, Astronomy, and Informatics, Nicolaus Copernicus University in Toruń, 87-100 Toruń, Poland
3 Molecular Sciences Institute, School of Chemistry, University of the Witwatersrand, Johannesburg 2050, South Africa
4 Institute of Advanced Studies, Nicolaus Copernicus University in Toruń, Ul. Wileńska 4, 87-100 Toruń, Poland
5 Department of Applied Sciences and Humanities, Faculty of Engineering and Technology, Jamia Millia Islamia, New Delhi - 110025, India

Table S1. Geometrical parameters of the IO_4^-···O–X contacts identified from the Cambridge Structural Database (CSD) (version 6.01). The search employed an I···O distance criterion of 2.0–4.0 Å and an O–I···O angular criterion of 140–180°. Only single-crystal structures with R-factor ≤ 0.05 and no reported crystallographic errors were considered. The table lists the CSD refcodes together with the corresponding I···O distances, *r*, and O–I···O angles, ∠.

CSD Refcode	∠O–I–O)/°	*r*(I···O)/Å
BEKNIS	160.543	3.952
BEKNIS	175.851	3.33
BEKNIS	169.773	3.94
BEKNOY	176.983	3.442
BEKNOY	162.314	3.621
BEKNOY	149.079	3.966

BEKNUE	140.216	3.819
BEKNUE	175.049	3.49
BEKNUE	141.163	3.91
BEKNUE	176.012	3.506
BEKPAM	162.545	3.516
BEKPAM	167.775	3.794
HOHMOG05	174.225	3.672
HOHMOG05	160.794	3.745
IHEHAG	175.436	3.616
JOJYOY	171.947	3.345
JOJYOY	161.11	3.804
JOJZAL	172.644	3.357
JOJZAL	161.047	3.842
LANNEU	177.161	3.26
LANNEU	172.227	3.105
LANNEU	169.137	3.586
TEJBET	165.93	3.704
TEJBET	174.755	3.482
TEJBET	164.57	3.714
TEJBET	178.572	3.339
TEJBET01	174.067	3.483
TEJBET01	164.317	3.729
TEJBET01	165.515	3.709
TEJBET01	178.894	3.345
TEJBIX	172.978	3.461
TEJBIX	177.13	3.316
TEJBIX	164.485	3.704
TEJBIX	165.529	3.705
TEJBIX01	173.256	3.524
TEJBIX01	165.539	3.734
TEJBIX01	165.939	3.744
TEJBIX01	177.281	3.411
WEMSUD	165.864	3.822
YIVYUB	173.007	3.527
YIVYUB	166.758	3.708
YIVYUB	166.901	3.721
YIVYUB	176.658	3.425
YOFBAA	175.301	3.376
YOFBAA	161.871	3.878
YOFBAA	175.174	3.386
YOFBAA	161.832	3.875
YOFBAA	161.854	3.877
YOFBAA	175.26	3.372

Table S2. Statistical summary of the $IO_4 \cdots O{-}X$ contacts identified from the Cambridge Structural Database (CSD). The search employed an I···O distance criterion of 2.0–4.0 Å and an O–I···O angular criterion of 140–180°. Only single-crystal structures with R-factor ≤ 0.05 and no reported crystallographic errors were included. The dataset comprises 49 close contacts extracted from 16 unique crystal structures.

Parameter	Number of Contacts	Minimum	Maximum	Mean	Standard Deviation
I···O Distance (Å)	49	3.105	3.966	3.606	0.214
O–I···O Angle (°)	49	140.2	178.9	168.2	8.6
